# Genomic Sequence and Virulence of Clonal Isolates of Vaccinia Virus Tiantan, the Chinese Smallpox Vaccine Strain

**DOI:** 10.1371/journal.pone.0060557

**Published:** 2013-04-12

**Authors:** Qicheng Zhang, Meijuan Tian, Yi Feng, Kai Zhao, Jing Xu, Ying Liu, Yiming Shao

**Affiliations:** 1 State Key Laboratory for Infectious Disease Prevention and Control, National Center for AIDS/STD Control and Prevention (NCAIDS), Chinese Center for Disease Control and Prevention, Beijing, China; 2 Key Laboratory of Molecular Microbiology and Biotechnology (Ministry of Education) and Key Laboratory of Microbial Functional Genomics (Tianjin), College of Life Sciences, Nankai University, Tianjin, China; 3 Division of Infectious Diseases & HIV Medicine, Case Western Reserve University, Cleveland, Ohio, United States of America; 4 National Vaccine and Serum Institute, Beijing, China; National Center for Biotechnology Information (NCBI), United States of America

## Abstract

Despite the worldwide eradication of smallpox in 1979, the potential bioterrorism threat from variola virus and the ongoing use of vaccinia virus (VACV) as a vector for vaccine development argue for continued research on VACV. In China, the VACV Tiantan strain (TT) was used in the smallpox eradication campaign. Its progeny strain is currently being used to develop a human immunodeficiency virus (HIV) vaccine. Here we sequenced the full genomes of five TT clones isolated by plaque purification from the TT (752-1) viral stock. Phylogenetic analysis with other commonly used VACV strains showed that TT (752-1) and its clones clustered and exhibited higher sequence diversity than that found in Dryvax clones. The ∼190 kbp genomes of TT appeared to encode 273 open reading frames (ORFs). ORFs located in the middle of the genome were more conserved than those located at the two termini, where many virulence and immunomodulation associated genes reside. Several patterns of nucleotide changes including point mutations, insertions and deletions were identified. The polymorphisms in seven virulence-associated proteins and six immunomodulation-related proteins were analyzed. We also investigated the neuro- and skin- virulence of TT clones in mice and rabbits, respectively. The TT clones exhibited significantly less virulence than the New York City Board of Health (NYCBH) strain, as evidenced by less extensive weight loss and morbidity in mice as well as produced smaller skin lesions and lower incidence of putrescence in rabbits. The complete genome sequences, ORF annotations, and phenotypic diversity yielded from this study aid our understanding of the Chinese historic TT strain and are useful for HIV vaccine projects employing TT as a vector.

## Introduction

Poxviruses are cytoplasm-replicating large double-stranded DNA viruses. Two of the best-known members of the family of *Poxviridae* are variola virus, the causative agent of smallpox, and vaccinia viruses (VACV), the smallpox vaccine strain. Smallpox was declared eradicated in 1979 by the World Health Organization (WHO) following a long, global vaccination campaign [1]. Despite smallpox eradication, studies on VACV remain relevant today. There is a continuing need for surveillance for potential smallpox outbreaks and bioterrorism acts using variola virus or other orthopoxviruses (OPVs) [2], [3], [4]. Furthermore, VACV has been employed to develop effective monkeypox vaccines [5], [6], [7]. The most widely used smallpox vaccine strain in China is the Temple of Heaven strain (also known as the Tiantan strain or VACV-TT). Its comparative strains are EM-63 in Russia, Lister/Elstree in Europe, New York City Board of Health (NYCBH)/Dryvax/Wyeth in the United States [8]. The most frequently studied VACV strain is Western Reserve (WR), a mouse brain passaged derivative of NYCBH [9], [10]. Virulence attenuation and immunogenicity improvement are important aspects of VACV based vaccine development [7], [11], [12].

Currently, there are 30 VACV genomes stored in the Viral Bioinformatics Resource Center (http://www.virology.ca). Several reports have illustrated the presence of genomic diversity in these earlier generations of smallpox vaccines. Qin et al., sequenced 11 clones isolated from Dryvax and revealed the presence of genetic diversity, with deletions in the right-hand inverted terminal repeat (ITR) [13]. Esposito et al., reported the presence of 573 single base polymorphisms and 53 insertions and deletions between the NYCBH/Dryvax clone Acam2000 and a more neurovirulent sister clone [14]. Garcel et al., showed that VACV-Lister also contained genotypic and phenotypic diversity [15] and shotgun sequencing of the genome of an unpurified Lister stock yielded more than 1,200 polymorphic sites [16].

The original TT strain was previously reported to have been isolated from skin lesions of a Chinese individual with smallpox in 1926 and had an attenuation history of passages in monkey, rabbit, bovine skin and rabbit testis [17]. TT had similar vaccination reaction characteristics as the contemporary Japanese cowpox strain and was used in the production of smallpox vaccine in China [17], [18], [19]. Originally, the TT vaccine was prepared from skin lesion materials after intracutaneous inoculations in calves. From 1969, it was produced in chicken embryo fibroblasts (CEF) cells and the TT (752-1) was a viral batch produced in 1975. The genomic sequence of a TT vaccine stock was determined and submitted to GenBank (accession number AF095689.1) in 1998. It was subsequently recognized that several sequencing errors existed [20]. Currently, an HIV candidate vaccine, rTV, uses TT as its vector [21]. It had completed a Phase I clinical trial and began a Phase II trial in 2012 (www.IAVI.org). In this study, we report the results from sequencing and virulence characterization of TT clones derived from TT (752-1).

## Materials and Methods

### Cells, Viruses, and Viral DNA Isolation

TT (752-1) was provided by the National Vaccine and Serum Institute, Beijing. NYCBH strain was provided by the AIDS Research and Reference Reagent Program (Catalog number 3929) of the U.S. National Institutes of Health. Both viruses were grown in CEF prepared from 9 to 11 day-old embryonic chicken eggs and cultured in Eagle’s medium supplemented with 10% fetal bovine serum, 1% L-glutamine, and 1% antibiotic at 37°C in a 5% CO_2_ atmosphere. TT clones were isolated from TT (752-1) by plaque-purifications at terminal dilutions. Seven viral plaques were isolated and amplified by sequential four rounds of passages to produce enough material for sequencing [13]. Plaque images (Fig. S1) were processed with ImageJ (National Institutes of Health) [22]. Genomic DNA of each clone was extracted and purified using proteinase K digestion followed by phenol-chloroform extraction as described previously [23].

### Genome Sequencing, Assembly, and Annotation

The genomes of TT clones were sequenced by Illumina Hiseq2000 Sequencer (Illumina Inc, CA) using shotgun sequencing of 500 bp paired-end sequencing library. Contigs were assembled and scaffolds were constructed using raw data with SOAP denovo and SOAP aligner (Beijing Genomics Institute) [24]. GenBank BLAST searches were performed for all scaffolds and sequences derived from the host genome were removed. The resulting alignments were analyzed by BioEdit (Ibis Bioscience, CA) and Contigexpress (Informax Inc, MD) to identify gaps between scaffolds. The average size of sequencing reads was 90 bp. Sequence gaps were present in the genome especially in ITRs. They were subsequently filled by polymerase chain reaction and Sanger sequencing.

Genome Annotation Transfer Utility (GATU) [25] was used to analyze ORFs in TT genomes using VACV-Copenhagen (Cop) as the reference. Orthologs and unassigned ORFs (i.e., ORFs that had no ortholog in Cop) were identified. ORFs shorter than 50 amino acids (aa) or without orthologous gene were excluded from the analysis. Unassigned ORFs of 50 aa or longer was categorized as OPVs genes according to the GenBank BLAST result with the highest similarity [26]. Viral Genome Organizer [27] was used to analyze the positions of individual genes. Sequences of ORFs with polymorphisms including frame-shift insertion or deletion among TT clones were further confirmed by Sanger sequencing**.** In cases where an ORF contained more than one start codon (ATG) near the 5′ terminal, BLAST was conducted to compare the orthologous sequences in other VACVs. The most frequently used ATG was selected as the tentative ORF in TT.

### Phylogenetic Analysis

BioEdit was used to produce alignments of multiple genomes by Clustal W method [28], and Base-By-Base software (http://athena.bioc.uvic.ca/virology-ca-tools/base-by-base/) [29], [30] was used to trim the alignments and produce a visual summary of whole genome alignments. Phylogenetic analysis was carried out using the alignments of the conserved central region of 160.4 kb DNA sequences between the two ITRs starting from TT_025 and ending with TT_255. This region in TT corresponded with the orthologous region between C7L and B16R in Cop. Global gaps (the gap regions present in more than half of these aligned genomes) [31] were removed from the alignments. Phylogenetic analysis was performed using a maximum-likelihood analysis with general time-reversible (GTR) substitution model, subtree-prune-and-regraft (SPR) improvement, and 1000 bootstrap replicates (http://www.atgc-montpellier.fr/phyml/). The phylogenetic tree was visualized using Figtree (http://tree.bio.ed.ac.uk/software/figtree/). MEGA 5 [32] was used to calculate pairwise distances between genomes by a Kimura 2-parameter substitution model including transition and transversion substitutions and pairwise-deletion treatment. The average values of pairwise distances between TT or Dryvax clones were calculated to illustrate the sequence diversity within these two groups of viruses. The standard deviation (σ) of each group was computed.

### Genomic Sequence Orthology

To analyze the genomic sequence orthology among TT clones, a consensus sequence was first derived. TT11 had the highest similarity to the consensus sequence and was therefore chosen as the reference. ORFs of TT11 were compared with orthologous ORFs of four other TT clones and other VACV genomes to calculate the full-length nucleotide sequence similarity using GATU and DNAMan (Lynnon Corporation, Quebec, Canada).

### Nucleotide Sequence Accession Numbers

The full genomic sequences of five TT clones were deposited in GenBank with accession numbers from JX489135 to JX489139. The OPVs used in this report and their GenBank accession numbers are listed in Table S1. TT-TW, a variant of TT (752-1) without plaque-purification was sequenced in this laboratory in 2010 (unpublished).

### Mouse Brain Based Neurovirulence Determination

TT viruses and the NYCBH strain were serially diluted in phosphate buffered saline (PBS) and then 3 x 10^2^ plaque forming units (PFU) in 30 µl PBS were inoculated intracranially to groups of five 3-week old female BALB/c mice anesthetized with 1.25% 2,2,2-tribromoethyl alcohol. Body weights and mortality were recorded daily during the 12-day observation period.

### Rabbit Skin Virulence Determination

TT clones and the NYCBH strain were serially diluted from 10^6^ to 10^2 ^PFU/ml in PBS. For each viral dilution, two rabbits were tested. Each rabbit received two dorsal skin intradermal inoculations with 0.1 ml viral preparation per injection site. At day 4 post-infection (p.i.), the diameters of lesions were measured and the incidence of putrescence was recorded.

### Ethics Statement

All experiments were conducted in accordance with the guidelines of the Laboratory Animal Center of Chinese Center for Disease Control and Prevention and NCAIDS. All procedures involving animal use and care were approved by the Institutional Committee on Laboratory Animals of NCAIDS.

## Results

### Clone Isolation and Genome Assembly

Seven randomly selected TT clones were isolated by plaque picking at the terminal dilution on CEF cells and amplified. The sizes of plaques varied after initial runs of plaque picking of each clone. No obvious difference was observed between the plaque sizes of the clones following passage and those of the original vaccine pool, although the average plaque sizes of TT9 and TT11 were significantly smaller (p<0.05) than those of TT7 and TT8 (Fig. S1).

All seven viral genomic DNAs were sequenced and their contigs were assembled. All the assemblies were aligned with Cop and ListerV107 to accurately identify any gaps, particularly in highly repeated elements such as the ITRs. Five TT clones (TT8 to TT12) were successfully assembled and generated full genomic sequences of 189,366 to 191,144 bp. Two clones, TT6 and TT7 had small gaps in the two ITRs and thus did not yield full genomic sequences.

### Phylogenetic Analysis

**Figure 1:**
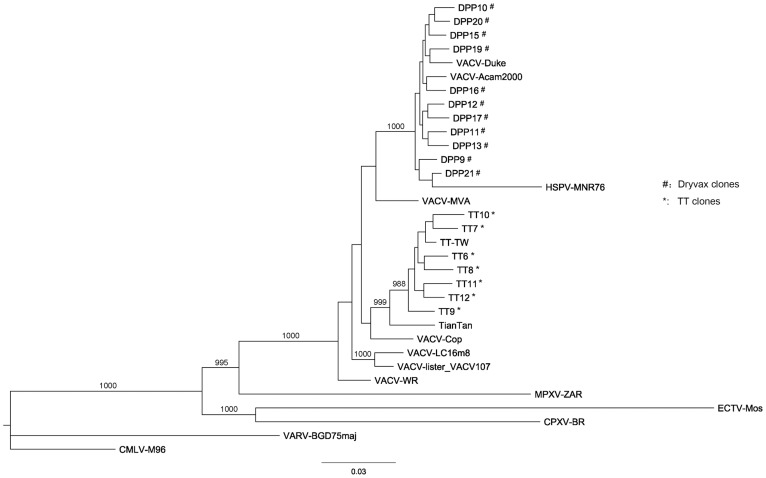
Phylogenetic analysis of TT and other OPV strains. Phylogenetic relationships between TT-associated strains (including Tian Tan and TT-TW), TT clones (marked by *), and OPVs including 11 Dryvax clones (marked by #). Bootstrap values higher than 900 are shown. Names and GenBank accession numbers of the viral strains shown in this figure are listed in Table S1. The scale bar of branch length was shown under the phylogenetic tree.

We examined the phylogenetic relationship of various OPVs by maximum-likelihood method using alignments of the conserved 160.4 kb central region (i.e., the ITR sequences were excluded) (Fig.1). These sequences included five TT plaque-purified clones, TT-TW, and 25 other commonly known OPVs including 11 Dryvax clones DPPs (Table S1). Although TT6 and TT7 clones contained incomplete ITRs, the regions used in phylogenetic analysis were complete and thus were included in this analysis.

Notably, TT clones clustered with a bootstrap value of 988 and shared a common ancestry with the TT genomic strain (AF095689.1) published in 1998 and another TT (752-1) derived TT-TW sequenced in 2010. Among the other VACVs, Cop was most similar to the TT cluster and HSPV-MNR76, NYCBH/Dryvax-derived DPP clones, Acam2000 and Duke exhibited least similarity. Among the analyzed OPV sequences, ectromelia virus strain Moscow (ECTV-Mos) was most distant from the TT cluster.

All the Dryvax derived strains clustered as one group, including the Dryvax clones (DPPs), Acam2000 that were independently isolated from another stock of the same vaccine, and Duke that isolated from a patient experiencing a Dryvax vaccine-associated complication. The TT clones exhibited higher sequence diversity than the Dryvax clones. The average value of pairwise distances between TT clones was 0.00401 (σ = 0.00026) and the average value between Dryvax clones was 0.00294 (σ  = 0.00017).

### Gene Annotations and Polymorphisms in Nucleotide Sequence

ORFs longer than 50 aa were initially annotated as genes and those without comparable orthologs in other OPVs were removed from annotation. Small ORFs that embedded in larger ORFs in the genomes of other poxviruses and those found to be truncated or split into two or more portions were removed from the ORF list and reclassified as “fragments”. Six such fragments were identified: TT_005.1, TT_028.1, TT_191.1, TT_210.1, TT_252.1, and TT_265.1. The genomic organizations of TT clones were identical to other sequenced VACVs. There were 255 unique ORFs in the genome and nine duplicated ORFs (TT_001 to TT_009 and TT_261 to TT_269) in ITRs. There are 273 ORFs found in each of the TT clones (Table S2), including orthologs of four additional conserved ORFs which were recently recognized in Cop and WR. These conserved ORFs have a “.5″ in their names.

**Figure 2:**
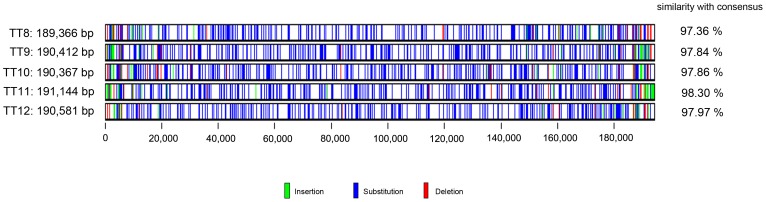
Distribution of polymorphic sites in TT genomes. The genomes were aligned by BioEdit and edited using Base-by-Base software. A consensus sequence was generated from TT8 to TT12 by Base-By-Base software. Sites in TT clones different from the consensus sequence are displayed in blue for nucleotide substitutions, green for insertions, and red for deletions. The extent of similarity (%) of each clone with the consensus sequence is displayed.

The orthology between TT clones was further assessed through the distribution of polymorphic sites. We first generated a consensus sequence using the most frequent nucleotide from each position. The similarities of each TT clone varied from 97.36% to 98.30% and TT11 had the highest similarity with the consensus (Fig. 2). The ORFs derived from TT11 were thus used as the reference for full-length ORF comparisons among TT clones (Table S3). Among the 273 annotated ORFs, the DNA sequences of 241 ORFs in four other TT clones had >95% identity with those in TT11. Conserved ORFs were located in the middle of the viral genome with gene functions related to viral replication and structure. For the remaining 32 ORFs, there was at least one clone displaying <95% identity with the reference ORF. More than 95% of the polymorphic ORFs exhibited length variations due to the acquisition of one or more point mutations, in-frame insertions or deletions. ORFs with relatively low similarity were located near ITRs where many virulence or immunomodulation-associated proteins reside.

Table 1 summarizes the major ORF differences among TT clones and four reference VACVs including Cop, ListerV107, WR, and Acam2000. Four ORFs (TT_147, TT_185, TT_188, and TT_190) in the TT genomic sequences had orthologs in TT genome (AF095689.1) and thus were annotated, but corresponding sequences in the reference genomes were not annotated. Whether these ORFs code for functional proteins remains to be investigated. Two ∼4 kb regions in TT strains (ORF TT_011 to TT_019 and TT_184 to TT_190, encoding host defense modulators, ankyrin-like proteins, and A-type inclusion protein) had orthologous regions in Acam2000 and WR, but were absent in Cop. Conversely, the regions coding three ORFs (C19L, C20L and C21L) in the ITRs of Cop were absent in TT genomes.

**Table 1:** Gross differences in ORF size (nt) and complement.

			Gene Size (bp)								
Gene Name	Function Description	Orthologs	TT8	TT9	TT10	TT11	TT12	Cop	ListerV107	Acam 2000	WR
VACV_TT_001 (ITR)^a^	Chemokine binding protein	VACWR001/218, Cop-C23L/B29R	720	726	720	735	729	735	777	726	735
VACV_TT_003 (ITR)	TNF alpha receptor (CrmB)	VACWR004/215, Cop-C22L/B28R	438	369	438	360	429	369	369	369	369
VACV_TT_004 (ITR)	Ankyrin-like protein	Cop-C18L/B24R	360	360	363	453	597	453	438	597	
VACV_TT_005 (ITR)	Ankyrin-like protein	Cop-C17L/B23R	540	735	1134	549	1140	1161	1275	1149	
VACV_TT_005.1 (ITR)	Ankyrin-like protein	Cop-C17L/B23R				492					
VACV_TT_006 (ITR)	Unknown ^b^	Cop-C16L/B22R	546	546	546	546	546	546	444	546	
VACV_TT_007 (ITR)	Unknown	Cop-C15L/B21R	210	210	210	210	210	276	270	270	
VACV_TT_008 (ITR)	EGF growth factor	VACWR009/210, Cop-C11R	423	426	423	426	426	429	423	420	423
VACV_TT_009 (ITR)	IL-1 receptor antagonist	VACWR010/209, Cop-C10L	996	996	996	996	996	996	996	996	996
VACV_TT_011	Zinc finger-like protein, apoptosis, host defense modulator	VACWR011/208, VARBSH-D6R/005 ^c^	252	252	363	153	252		543	252	546
VACV_TT_012	Zinc finger-like protein, apoptosis, host defense modulator	VACWR012/207, VARBSH-D6R/005	189	189	189	189	189		201	189	189
VACV_TT_013	Soluble IL-18 binding protein (Bsh-D7L), host defense modulator	VACWR013, VARBSH-D7L/006	381	381	381	381	381		381	375	381
VACV_TT_014	Ankyrin-like protein (Bang-D8L), 77 kDa cowpox host-range protein	VACWR014, CPXVBR-V024 ^d^	273	273	273	273	273			273	714
VACV_TT_015	Ankyrin-like protein (Bang-D8L), 77 kDa cowpox host-range protein	VACWR014, CPXVBR-V024	429	429	933	429	429		429	429	
VACV_TT_016	Ankyrin-like protein (Bang-D8L), 77 kDa cowpox host-range protein	VACWR015, CPXVBR-V024	192	426		414	399			408	414
VACV_TT_017	Ankyrin-like protein (Bang-D8L), 77 kDa cowpox host-range protein	VACWR016, CPXVBR-V024	234	234	234	234	234		234	234	234
VACV_TT_018	Ankyrin-like protein (Bang-D8L), 77 kDa cowpox host-range protein	VACWR017, CPXVBR-V024	216	216	216	216	216			216	216
VACV_TT_019	Unknown	VACV-Lister_VACV107-List015	180	180	180	180	180		180		
VACV_TT_020	Ankyrin-like protein	VACWR019, Cop-C9L	1905	1905	1899	1905	1905	1905	1905	1905	1905
VACV_TT_022	Unknown	VACWR020, Cop-C8L	534	534	534	534	534	555	534	534	534
VACV_TT_025	Host range virulence factor	VACWR021, Cop-C7L	453	453	453	453	453	453	453	453	453
VACV_TT_026	Unknown	VACWR022, Cop-C6L	456	363	456	456	456	456	456	456	456
VACV_TT_027	Kelch-like protein	VACWR023, Cop-C5L	615	615	561	615	615	615	615	615	615
VACV_TT_028	IL-1 receptor antagonist	VACWR024, Cop-C4L	951	570	387	594	951	951	951	189	951
VACV_TT_028.1	IL-1 receptor antagonist	VACWR024, Cop-C4L			189						
VACV_TT_029	Secreted complement binding protein	VACWR025, Cop-C3L	792	786	786	792	792	792	792	792	792
VACV_TT_031	Kelch-like protein	VACWR026, Cop-C2L	1539	1539	1539	1539	1539	1539	1539	1521	1539
VACV_TT_032	Unknown	VACWR027, Cop-C1L	675	675	675	690	675	675	675	675	690
VACV_TT_035	Ankyrin-like protein	VACWR030, Cop-M1L	1413	1413	1413	1413	1413	1419	1413	1410	1419
VACV_TT_036	NF-κB inhibitor	VACWR031, Cop-M2L	663	663	663	663	663	663	663	663	663
VACV_TT_038	Serpin (serine protease inhibitor) (SPI-3)	VACWR033, Cop-K2L	1110	1110	1110	1110	1110	1110	1110	1110	1110
VACV_TT_041	IFN resistance, elF2 alpha-like PKR inhibitor	VACWR034, Cop-K3L	267	267	267	267	267	267	267	267	267
VACV_TT_043	Monoglyceride lipase	VACWR037, Cop-K5L	366	366	366	366	366	411	528	411	405
VACV_TT_044	Monoglyceride lipase	VACWR038, Cop-K6L	255	246	246	255	255	246	246	246	246
VACV_TT_046	Apoptosis inhibitor (mitochondrial-associated)	VACWR040, Cop-F1L	723	681	681	681	681	681	681	681	681
VACV_TT_054	Unknown	VACWR046, Cop-F7L	249	249	249	243	249	279	243	243	243
VACV_TT_059	Unknown	VACWR050, Cop-F11L	1065	1065	1065	1065	1065	1065	1065	1047	1047
VACV_TT_063.5	IMV protein	VACWR53.5	150	150	150	150	150				150
VACV_TT_065	Unknown	VACWR054, Cop-F15L	477	477	477	477	477	477	477	477	444
VACV_TT_071	IFN resistance, PKR inhibitor	VACWR059, Cop-E3L	573	573	573	573	573	573	573	573	573
VACV_TT_073	Virosome component	VACWR061, Cop-E5R	1026	1026	1026	1026	1026	996	996	570	1026
VACV_TT_080	DNA polymerase	VACWR065, Cop-E9L	3021	3021	3021	3021	3021	3021	3021	3018	3021
VACV_TT_086	Unknown	CPXVBR-078A	189	189	189	189	189				
VACV_TT_101.5	RNA polymerase (RPO7)	VACWR083, Cop-G5.5R	192	192	192	192	192	192	192	192	192
VACV_TT_102	Virulence factor, NIpC/P60 superfamily protein	VACWR084, Cop-G6R	498	498	498	498	498	498	498	498	498
VACV_TT_109	Unknown	VACWR089, Cop-L2R	258	258	258	264	258	264	264	258	264
VACV_TT_126	Unknown	CPXVBR-116	183	183	183	183	183				
VACV_TT_147	Unknown	VACV-TianTan-134	219	219	219	219	219				
VACV_TT_151.5	Thioredoxin-like protein, S-S bond formation pathway protein	VACWR121, Cop-A2.5L	231	231	231	231	231	231	231	231	231
VACV_TT_154	39kDa core protein	VACWR123, Cop-A4L	852	852	852	846	852	846	846	846	846
VACV_TT_162	IMV Membrane protein	VACWR128, Cop-A9L	300	327	312	327	327	300	300	327	327
VACV_TT_163	P4a (precursor of core protein 4a)	VACWR129, Cop-A10L	2679	2679	2679	2679	2679	2676	2676	2676	2676
VACV_TT_168	Structural protein	VACWR131, Cop-A12L	576	576	579	576	576	579	579	579	579
VACV_TT_170.5	IMV membrane protein, Virulence factor	VACWR134, Cop-A14.5L	162	162	162	162	162	162	162	162	162
VACV_TT_180	Holliday junction resolvase	VACWR142, Cop-A22R	564	564	564	531	564	531	564	564	564
VACV_TT_184	Cowpox A-type inclusion protein	VACWR146	639		720	639	720			465	465
VACV_TT_185	Unknown	VACV-TianTan-171	207	207		207	207				
VACV_TT_186	Cowpox A-type inclusion protein	MPXV_USA2003_044_143 ^e^	162	162	162	162	162				
VACV_TT_187	Cowpox A-type inclusion protein	VACWR147	693	693	684	684	693			684	684
VACV_TT_188	Unknown	VACV-TianTan-173	186	186	186	186	186				
VACV_TT_189	Cowpox A-type inclusion protein	VACWR148	2178	2178	2178	2178	2178			2166	2178
VACV_TT_190	Unknown	VACV-TianTan-175	183	183	183	183	183				
VACV_TT_191	P4c (precursor of core protein 4c)	VACWR149, Cop-A26L	1509	1509	1509	609	1509	969	1509	1503	1503
VACV_TT_191.1	P4c (precursor of core protein 4c)	VACWR149, Cop-A26L				846					
VACV_TT_198	ATPase, DNA packaging protein	VACWR155, Cop-A32L	903	813	813	813	813	903	903	813	813
VACV_TT_207	Unknown	VACWR161, VARGAR-A43R ^f^	195	195	195	195	195		189	189	189
VACV_TT_208	Unknown	VACV-REC_GLV_1h68-225^g^	183	183	183	183	183				
VACV_TT_210	Semaphorin	VACWR163, Cop-A39R	1212	687	687	774	774	1212	1212	399	888
VACV_TT_210.1	Semaphorin	VACWR164		429	468	468	468			429	429
VACV_TT_212	C-type lectin-like type-II membrane protein	VACWR165, Cop-A40R	480	480	480	480	480	507	480	507	480
VACV_TT_213	Secreted virulence factor	VACWR166, Cop-A41L	660	660	660	660	660	660	660	660	660
VACV_TT_216	Unknown	VACWR169	237	237	237	237	237		237		237
VACV_TT_217	Hydroxysteroid dehydrogenase	VACWR170, Cop-A44L	1041	1041	1041	1041	1041	1041	1041	1041	1041
VACV_TT_219	IL-1 signaling inhibitor	VACWR172, Cop-A46R	723	723	723	723	723	645	723	723	723
VACV_TT_221	Unknown	VACWR173, Cop-A47L	759	759	759	759	735	735	759	735	759
VACV_TT_222	Thymidylate kinase	VACWR174, Cop-A48R	684	684	684	684	684	615	684	615	684
VACV_TT_228	Intracellular TLR and IL-1 signal inhibitor	VACWR178, Cop-A52R	573	573	573	573	573	573	573	573	573
VACV_TT_229	Unknown	Cop-A54L	273				273	273	258		
VACV_TT_230	TNF receptor (CrmC)	VACWR179, Cop-A53R	399	399	561	561	399	312	561	399	312
VACV_TT_231	Unknown	VACV-Lister_VACV107-173	180	180	180	180	180		180		
VACV_TT_232	Kelch-like protein	VACWR180, Cop-A55R	1695	1695	1695	1695	1695	1695	1695	1695	1695
VACV_TT_242	Complement control, CD46, EEV	VACWR187, Cop-B5R	954	954	954	954	954	954	666	954	954
VACV_TT_247	Unknown	VACV-REC_GLV_1h68-269	174	174	174	174	174				
VACV_TT_250	Unknown	VACWR193, Cop-B11R	231	231	231	228	219	267	219	219	219
VACV_TT_252	Serpin 1,2,3	VACWR195, Cop-B13R, Cop-B14R	1008	351	1008	1008	1008	351	351	381	1038
VACV_TT_252.1	Serpin 1,2,3	VACWR195, Cop-B13R, Cop-B14R		669				669	669	669	1038
VACV_TT_255	IL-1 beta receptor	VACWR197, Cop-B16R	873	873	873	873	873	873	981	981	981
VACV_TT_258	Ankyrin-like protein	VACWR199, Cop-B18R	1725	1725	1725	1725	1725	1725	1242	1725	1725
VACV_TT_259	IFN alpha/beta receptor	VACWR200, Cop-B19R	1056	1062	1056	1056	1062	1062		798	1056
VACV_TT_260	Ankyrin-like protein	Cop-B20R	1809	1794	441	1794	1842	384			
VACV_TT_261 (ITR)	IL-1 receptor antagonist	VACWR010/209, Cop-C10L	996	996	996	996	996			996	996
VACV_TT_262 (ITR)	EGF growth factor	VACWR009/210, Cop-C11R	423	426	426	426	426			420	423
VACV_TT_263 (ITR)	Unknown	Cop-C15L/B21R	210	210	210	210	210	276			
VACV_TT_264 (ITR)	Unknown	Cop-C16L/B22R	546	546	546	546	546	546	444	546	
VACV_TT_265 (ITR)	Ankyrin-like protein	Cop-C17L/B23R	540	735	1134	549	1140	1161	1275	1149	
VACV_TT_265.1 (ITR)	Ankyrin-like protein	Cop-C17L/B23R				492					
VACV_TT_266 (ITR)	Ankyrin-like protein	Cop-C18L/B24R	360	360	363	453	597	453		597	
VACV_TT_267 (ITR)	TNF alpha receptor (CrmB)	Cop-C22L/B28R	438	369	438	360	429	369	369	369	369
VACV_TT_268 (ITR)	Chemokine binding protein	VACWR001/218, Cop-C23L/B29R	720	726	720	735	729	735	777	726	735

Gross differences in ORF size (nt) and complement among TT clones and four VACVs (Cop, ListerV107, Acam 2000, and WR). a: ITR, inverted terminal repeat; b: genes with unknown functions; c: CPXVBR, Cowpox virus Brighton Red; d: VARBSH, Variola major virus Bangladesh; e: MPXV_USA2003_044, monkeypox virus strain USA2003_044; f: VARGAR, variola virus Garcia strain; g: VACV-REC_GLV_1h68, Vaccinia virus strain recombinant GLV-1h68 strain.

**Figure 3:**
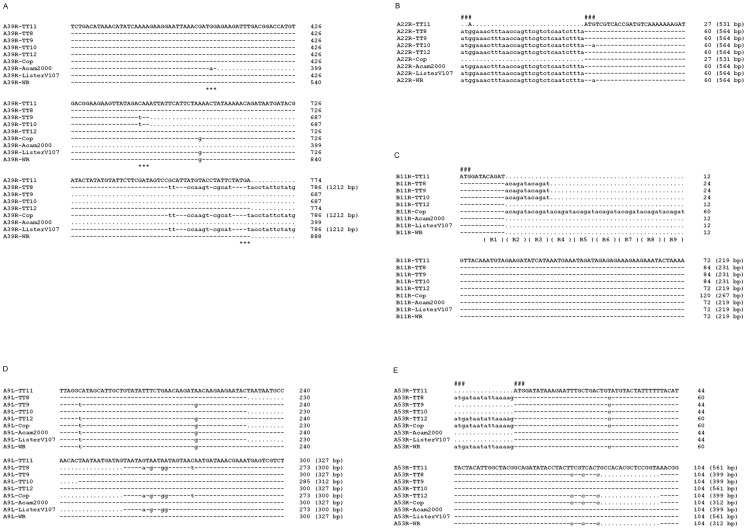
Representative nucleotide sequence polymorphisms in five ORFs among TT clones and four reference VACV genomes. Nucleotide sequence polymorphisms in ORFs TT_210/210.1 (A39R) (A), TT_180 (A22R) (B), TT_250 (B11R) (C), TT_162 (A9L) (D), and TT_230 (A53R) (E) are shown. The TT11 clone with the highest sequence similarity to the consensus sequence was used as the reference. Altered nucleotides are shown in lower case. Nucleotides identical to TT11 are denoted with “–” and nucleotides not present in TT11 or other strains are denoted with dots. ###: start codon, ***: stop codon, R: 6-bp nucleotide repeat, numbers in parenthesis: nucleotide length of the ORF.

Several genes had polymorphic lengths because of the presence of substitution mutations. As shown in Fig. 3A, the length of ORF TT_210/210.1 (A39R, also known as semaphorin) in TT8 was the same as that in Cop and ListerV107 (1212 bp). In TT9 and TT10, a C685T mutation truncated this ORF to 687 bp and a T774A mutation truncated this ORF to 774 bp in TT11 and TT12 or 888 bp in WR. In another example, shown in Fig. 3B, the start codons for ORF TT_180 (A22R, Holliday junction resolvase) in TT11 and Cop were altered by a T-A mutation near the 5′ terminal and the next available start codon was 33 bp downstream. This change shortens the size of this ORF to 531 bp. Several polymorphisms were generated by insertions or deletions. One interesting example was the addition of copies of 6 bp repeats (ACAGAT) in TT_250 (B11R, function unknown) (Fig. 3C). In TT11, TT12, Acam2000, ListerV107, and WR, there was one copy of ACAGAT and in TT8, TT9, and TT10 there were three copies. Nine copies were present in Cop. In addition, in-frame insertions near the 3′ terminal of TT_162 (A9L, intracellular mature virion membrane protein) resulted in ORFs of 327 bp in TT11, TT9, TT12, Acam2000, and WR; 312 bp in TT10; and 300 bp in TT8, Cop, and ListerV107 (Fig. 3D). TT_230 (A53R, TNF receptor CrmC) in TT8, TT12, Cop, and WR consisted of a 16 bp frame-shift deletion near the 5′ end and a shift of start codon 16 bp upstream from the ones in TT11, TT9, TT10, Acam2000, and ListerV107 (Fig. 3E).

### Polymorphisms in Virulence Reaction Associated Proteins

VACV contains more than 200 genes, some of which have been previously reported to be associated with viral virulence and immunomodulation. We selected seven virulence reaction-associated genes from TT clones and Acam2000 for amino acid sequence comparison (Table 2). Acam2000 was a clonal Vero cell culture vaccinia virus derived from NYCBH/Dryvax, and its relative gene sequences were selected to represent those of the NYCBH strain used in the our study. TT_213 (A41L) was a secreted virulence factor and its deletion was reported to result in severe lesions and increased viral clearance in the mouse skin model [33]. Six amino acid polymorphic sites were found in TT clones and Acam2000. TT clones displayed a substitution mutation, K122E, located in the chemokine binding protein superfamily conserved domain. The change from alkaline lysine (K) to glutamic acid (E) residue might be associated with the attenuation of TT. Seven polymorphic sites were found in TT_242 (B5R) among TT clones. B5R was a type I integral membrane glycoprotein and extracellular enveloped virion (EEV) complement control protein which blocked complement activation and associated inflammatory responses. Destruction of B5R was reported to contribute to attenuation of VACV [16], [34]. There were three substitution mutations among TT clones and Acam2000, L55S, V82I and I153M. L55S changed the polarity of the amino acid.

**Table 2:** Mutations identified in the seven virulence-associated proteins.

Gene Name	Protein Size (aa)	Protein Function (Ortholog in Cop)	Non-Synonymous site^a^	Acam2000	TT8	TT9	TT10	TT11	TT12
TT_025	150	Host range virulence factor	31	K ^b^	–	–	–	K31R	–
		(Cop-C7L)	38	D	–	–	–	D38G	D38G
			41	K	–	–	–	–	K41Q
			130	N	–	–	–	–	N130T
TT_102	165	Virulence factor, NIpC/P60,	90	N	N90D	N90D	N90D	N90D	N90D
		superfamily protein (Cop-G6R)	105	N	N105D	N105D	–	–	–
			115	E	E115D	E115D	E115D	E115D	E115D
			122	I	I122V	I122V	I122V	I122V	I122V
			123	D	D123E	D123E	D123E	D123E	D123E
TT_170.5	53	IMV membrane protein, virulence factor (Cop-A14.5L)	52	A	–	A52V	A52V	–	–
TT_213	219	Secreted virulence factor	5	L	–	L5V	L5V	–	–
		(Cop-A41L)	7	I	–	I7V	I7V	–	–
			47	H	–	H47P ^c^	H47P	H47P	H47P
			122	K	K122E	K122E	K122E	K122E	K122E
			160	D	–	–	–	–	D160N
			178	A	–	A178T ^c^	A178T	A178T	A178T
TT_242	317	Complement control, CD46,	40	N	N40D	N40D	–	N40D	N40D
		EEV (Cop-B5R)	41	N	N41K	N41K	–	N41K	N41K
			55	L	L55S ^c^	L55S	L55S	L55S	L55S
			82	V	V82I	V82I	V82I	V82I	V82I
			140	C	–	C140Y	–	C140Y	C140Y
			153	I	I153M	I153M	I153M	I153M	I153M
			216	I	–	–	–	I216T ^c^	I216T
TT_245	182	Virulence, ER resident	15	L	–	–	–	L15V	L15V
		(Cop-B7R)	104	R	R104K	R104K	–	R104K	R104K
			168	D	D168N	-	–	–	–
TT_248	77	Virulence factor (Cop-B9R)	54	R	R54L ^c^	-	–	–	–
			59	G	G59E	-	–	–	–

Mutations identified in the seven virulence-associated proteins in five TT clones and Acam2000. Acam 2000 is used as the reference. Amino acid (aa) in TT clones identical to the reference is shown as “–”. a: amino acid position in Acam2000; b: single letter aa code; c: aa mutation resulted in polarity change. Abbreviations, IMV: intracellular mature virion, EEV: extracellular enveloped virion, ER: endoplasmic reticulum.

**Figure 4:**
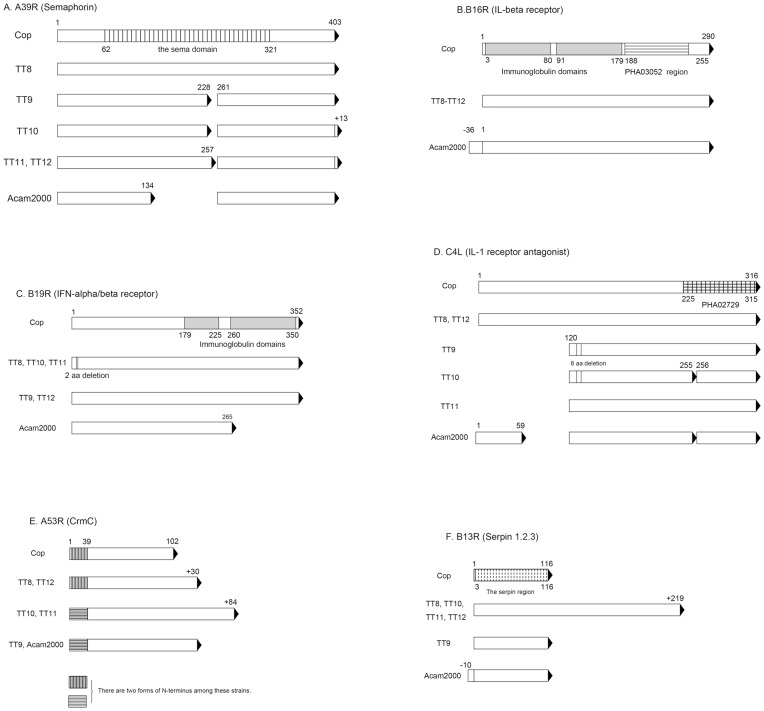
Major polymorphisms within immunomodulation-associated proteins in TT clones, Acam2000, and Cop. Six proteins previously known to be associated with immunomodulation were selected for comparison using Cop as the reference strain. These proteins are A39R encoding semaphorin (A), B16R encoding an IL-1 beta receptor (B), B19R encoding a soluble and cell-surface interferon- α/β receptor (C), C4L encoding an IL-1 receptor antagonist (D), A53R encoding TNF receptor CrmC (E), and B13R encoding serine protease inhibitors (Serpin 1, 2 and 3) (F). Special functional domains of proteins are depicted. Premature terminations are shown as solid triangles. Locations of insertions and deletions are also shown.

Six immunomodulation-associated genes were also selected for closer examination (Fig. 4). TT_210 (A39R) encoded a semaphorin influencing the outcome of dermal infection and, when truncated, the protein product could not be exported extracellularly [35]. Presence of a stop codon in the central sema domain yielded a truncated product of 228 aa in TT9 and TT10; and 257 aa in TT11 and TT12 (Fig. 4A). Truncation of the protein was also observed in Acam2000, with a product of 134 aa. TT_255 (B16R) encoded an IL-1 beta receptor. It was 290 aa in length in Cop and all five TT strains. It was longer in Acam2000 due to the presence of additional N-terminal 36 aa (Fig. 4B). TT_259 (B19R) encoded interferon-α/β receptor. In Cop, TT9, and TT12, it was 352 aa in length. There was a 2-aa deletion (Ser-Leu) in TT8, TT9, and TT10 near the N-terminus (Fig. 4C). In Acam2000, B19R was 265 aa and lost the immunoglobulin domain near the C-terminal. TT_028 (C4L) encoded IL-1 receptor antagonist and was 316 aa in length in Cop, TT8, and TT12 (Fig. 4D). It was shorter in TT9, TT10 and TT11 due to the deletion of 120 aa in N-terminal. In Acam2000, C4L was split into three fragments. TT_230 (A53R) encoded TNF receptor (CrmC) and its length differed greatly among these genomes (Fig. 4E). It was 102 aa in Cop and was 30 aa longer in TT8, TT9, TT12, and Acam2000. In TT10 and TT11, there was a 84 aa elongation in the C-terminal. Two forms of N-terminal sequences were present in A53R as described earlier. TT_252 (B13R) encoded serine protease inhibitors (Serpin 1, 2, 3) and was 116 aa in length in both Cop and TT9 (Fig. 4F). It was 335 aa in four other TT clones due to the presence of additional C terminal 219 aa. In Acam2000 it was 126 aa due to the presence of an additional N-terminal 10 aa.

### Virulence of TT Clones and NYCBH in Animal Models

**Figure 5:**
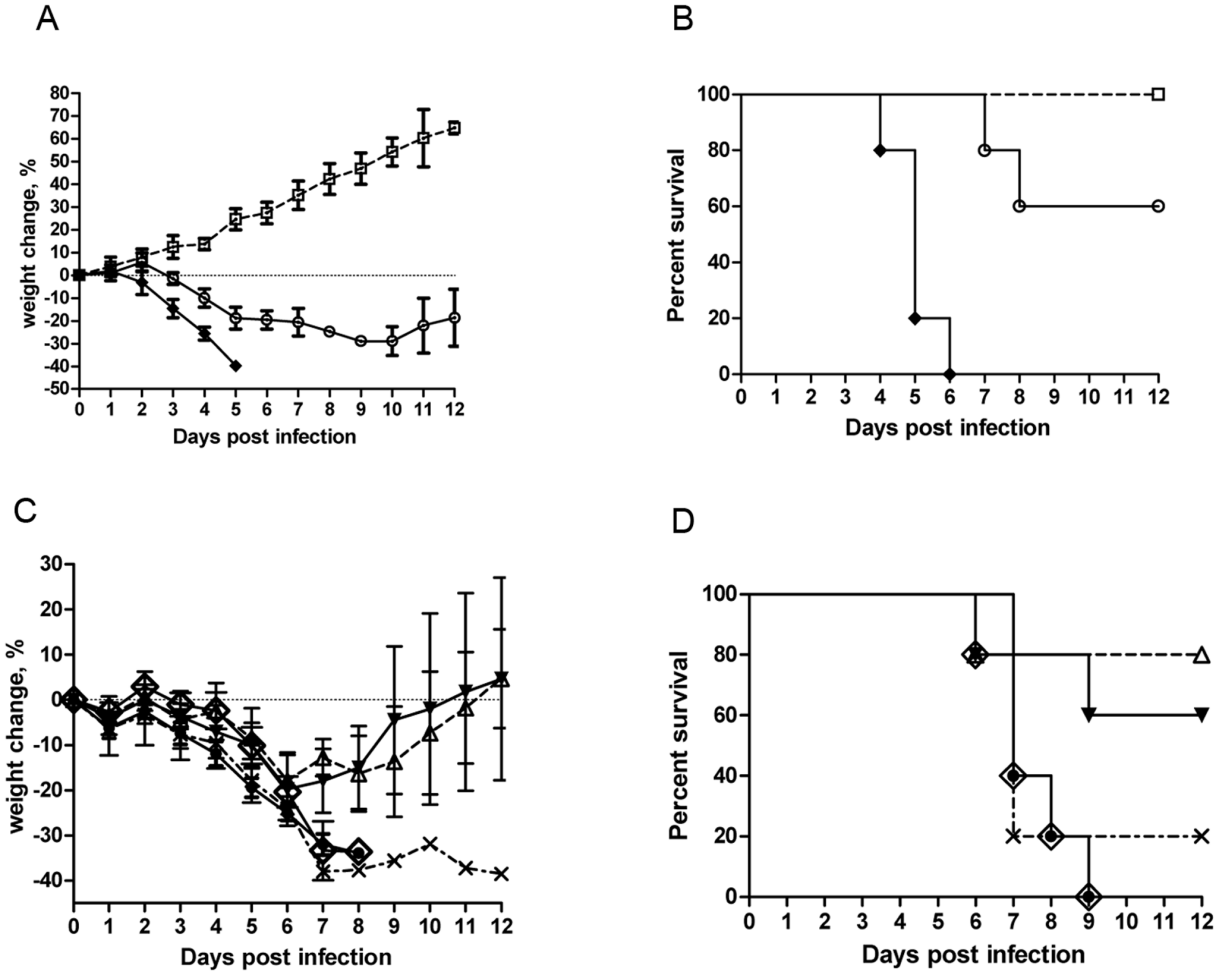
Viral neurovirulence properties in mice with intracranial inoculation. BALB/c mice (n = 5 per testing group) were inoculated with 3 x 10^2^ PFU of NYCBH, TT (752-1) or five TT clones (TT8 to TT12). Body weights and mortality were monitored daily for 12 days. Average body weight change (%) and one standard deviation (vertical bar) were calculated by comparing the body weight pre- and post-inoculation. Body weight change (A) and mortality (B) in TT (752-1), NYCBH, and PBS control; and body weight change (C) and mortality (D) in five TT clones. Symbols in (A) and (B): PBS: (□), TT (752-1): (○), NYCBH: (♦). Symbols in (C) and (D): TT8: (◊), TT9: (▾), TT10: (•), TT11: (▵), TT12: (×).

We compared the neurovirulence of parental TT (752-1), TT clones, and NYCBH. Groups of three-week-old BALB/c mice were intracranially inoculated with 3 x 10^2^ PFU viral preparations or PBS and observed daily for weight change and mortality. In the control group (injected with PBS), all mice gained weight over the 12-day observation period. Mice that received NYCBH showed severe signs of illness, weight loss (Fig. 5A), and decreased physical activity starting on day 3 p.i. One mouse died on day 4, three more died on day 5 (Fig. 5B). By day 5, the remaining mouse had lost 39.7% of its initial body weight and died at day 6. For the TT (752-1) group, there was an initial weight gain, but on day 3 the mice began showing weight loss that persisted to day 11. One mouse died on day 7, another on day 8. The remaining three mice survived and gained body weight. The weight change among mice infected with TT (752-1) was less than those infected with NYCBH (P<0.05).

We examined the virulence of five TT clones in the same manner. The patterns of weight loss (Fig. 5C) and mortality (Fig. 5D) were similar to their parental strain, but differences among clones were observed. Mice injected with TT8 or TT10 lost 33% of their body weight by day 8 and all of them died on day 9. One of the mice injected with TT12 lost 32% of its body weight, but survived to day 12. Three and four of the mice injected with TT9 and TT11, respectively, initially lost weight but survived and regained body weight up to the original level.

We further characterized the skin virulence of the parental and cloned strains by inoculating four viral preparations, 10^2^ to 10^5^ PFU, into rabbit dorsal skin. Viral virulence was quantified by the diameter of skin lesion on day 4 p.i. As shown in Table 3, among the viral strains tested, NYCBH produced the largest lesions with the largest lesion measuring 9.8 mm in diameter at 10^5^ PFU. Among TT strains, the parental strain yielded the largest lesions at the dilution of 10^5^ PFU (4.6 mm) followed by TT8 (4.2 mm), TT9 (3.4 mm), TT10 (3.4 mm), TT12 (1.2 mm), and TT11 (1.0 mm). NYCBH yielded 100% putrescence at all dilutions. TT clones yield 100% putrescence at the highest 10^5^ PFU inoculation, much less putrescence at the 10^4^ PFU inoculation, and no putrescence at the 10^3^ PFU inoculation.

**Table 3:** Viral skin virulence in rabbits.

	10^5^ PFU		10^4^ PFU		10^3^ PFU		10^2^ PFU	
VACV strain	Average diameter of lesion (mm)	Incidence of putrescence (%)	Average diameter of lesion (mm)	Incidence of putrescence (%)	Average diameter of lesion (mm)	Incidence of putrescence (%)	Average diameter of lesion (mm)	Incidence of putrescence (%)
TT8	4.2	100	3.0	50	0	0	0	0
TT9	3.4	100	0	0	0	0	0	0
TT10	3.4	100	1.7	75	0	0	0	0
TT11	1.0	100	0.8	50	0	0	0	0
TT12	1.2	100	0.9	25	0	0	0	0
TT (752-1)	4.6	100	4.1	50	0	0	0	0
NYCBH	9.8	100	6.7	100	5.2	100	4.0	100

Viral skin virulence in rabbits with intradermal inoculation. Rabbits were inoculated intradermally in dorsal skin with 10^2^ to 10^5^ PFU viral preparations from five TT clones, parental TT (752-1), and NYCBH. The average lesion diameter (mm) and putrescence incidence percentage are shown.

## Discussion

Similar to most vaccinia viruses used in the last century, TT has a vague origin and passage history. The earliest record shows its ancestral strain was isolated from skin lesions of a Chinese individual with smallpox in 1926 [17], [18], [19]. Vaccine stocks were established after monkey-rabbit-bovine passages and were later widely used in China during the smallpox eradication campaign. After 1969, TT vaccines were produced in CEF cell cultures but were never plaque-cloned. The lack or loss of well-organized records for vaccine production further add to the confusion of the identity and lineage of the virus [36]. Here we successfully characterized full genomes of five TT clones isolated from a CEF-derived stock, TT (752-1). Sequences from two other clones with incomplete terminal ITRs were also obtained. Our study, analogous to an earlier genetic analysis [37], shows that TT was a vaccinia virus, not a variola virus. Phylogenetic tree analysis shows that all TT clones were a mixture of closely clustered quasi-species with an inter-clone diversity slightly higher than that observed in clones derived from NYCBH/Dryvax. Presence of quasi-species was also reported in Lister/Elstree vaccine strains [12], [16], [38].

The genome structure of TT is the same as other vaccinia viruses. The genome length of TT clones varied from 189,366 in TT8 to 191,144 in TT11 with 273 identifiable ORFs. Insertions, deletions, and point mutations were present throughout the genome resulting in protein alterations. ORFs located in the middle of the genome were more conserved than those located at the two ITRs. We also examined biological activities of TT strains in mice using intracranial injections. TT (752-1) and its progeny clones behaved different in the induction of weight loss and mortality. These biological variations among TT clones may not be due to the replication abilities since they were similar in our study (data not show). The severity of the weight loss and mortality was less than that produced by the NYCBH strain. Our finding differs from Marennikova et al. who reported that TT displayed higher pathogenicity than NYCBH in mice [39], [40]. The TT stock used by Marennikova et al., was calf skin-derived, not one of the CEF-derived viral strains generated after 1969. We did not examine a calf skin-derived strain in our study and thus direct comparison between the calf skin and CEF derived TT viruses could not be made. Using NYCBH as the common denominator in both studies, it appears passage in CEF might have attenuated the virulence of the calf skin-derived TT. Likewise, we found TT strains displayed less virulence than NYCBH in mice and rabbits. This finding is corroborated by historic data. The frequency of post vaccination encephalitis from CEF-derived TT was 2.1 cases/million for primary vaccinations and 0 for progressive vaccinia; the corresponding data from calf skin-derived NYCBH were 2.9∼12.3 and 0.9∼1.5, respectively [8], [41]. Further systemic genotypic and phenotypic comparison might reveal underlying mechanisms related to viral virulence and immunomodulation.

Our present genomic and phenotypic characterization of TT strains yields valuable information for continuing TT-based research in China or elsewhere to produce safer and more effective smallpox vaccines. It is also useful for the current vaccine research using TT-based vectors for HIV and other pathogenic viruses.

## Supporting Information

Figure S1 **Plaque properties of TT (752-1) and seven TT clones.** Each clone of the TT (752-1) was plaque-purified and ∼50 PFU of each clone was plated on a monolayer of CEF cells. The infected cells were cultured for 3 days and then stained with crystal violet and imaged. The plaques were visualized using ImageJ and their sizes were determined.
(TIF)

Table S1 Identities and GenBank accession numbers of orthopoxviruses cited in this work.
(DOC)

Table S2 **Complete complement of genes and gene fragments encoded by TT clones.** a: The orthologs in Cop and WR were used as the reference. When no ortholog was present in these two reference strains the gene with the highest similarity in OPVs was used. b: orthologs of "X_ORF_X" in Cop were annotated in TT clones. These genes were predicted not to express. c: TT_005.1, TT_028.1, TT_191.1, TT_210.1, TT_252.1, and TT_265.1 were identified as fragments.
(DOC)

Table S3 **Full length similarities among nucleotide sequences of orthologs in TT clones and four reference strains to TT11.** TT11 was used as the reference clone for comparison with TT8, TT9, TT10, TT12, and four other VACVs (Cop, ListerV107, Acam2000 and WR).
(DOC)

## References

[1] The global eradication of smallpox: final report of the Global Commission for the Certification of Smallpox Eradication, Geneva, December 1979. Geneva, World Health Organization, 1980.

[2] Henderson DA (1999) The looming threat of bioterrorism. Science 283: 1279–1282. 10037590 10.1126/science.283.5406.1279

[3] Levy-Bruhl D, Guerin N (2001) The use of smallpox virus as a biological weapon: the vaccination situation in France. Euro Surveill 6: 171–178. 11891388 10.2807/esm.06.11.00385-en

[4] Mahalingam S, Damon IK, Lidbury BA (2004) 25 years since the eradication of smallpox: why poxvirus research is still relevant. Trends Immunol 25: 636–639. 15530831 10.1016/j.it.2004.10.002

[5] Earl PL, Americo JL, Wyatt LS, Espenshade O, Bassler J, et al (2008) Rapid protection in a monkeypox model by a single injection of a replication-deficient vaccinia virus. Proc Natl Acad Sci U S A 105: 10889–10894. 18678911 10.1073/pnas.0804985105 PMC2495015

[6] Stittelaar KJ, van Amerongen G, Kondova I, Kuiken T, van Lavieren RF, et al (2005) Modified vaccinia virus Ankara protects macaques against respiratory challenge with monkeypox virus. J Virol 79: 7845–7851. 15919938 10.1128/JVI.79.12.7845-7851.2005 PMC1143678

[7] Earl PL, Americo JL, Wyatt LS, Eller LA, Whitbeck JC, et al (2004) Immunogenicity of a highly attenuated MVA smallpox vaccine and protection against monkeypox. Nature 428: 182–185. 15014500 10.1038/nature02331

[8] Fenner F, Henderson DA, Arita I, Jezek Z, Ladnyi ID, et al. (1988) Smallpox and its eradication. Geneva, World Health Organization.

[9] Parker RF, Bronson LH, Green RH (1941) Further Studies of the Infectious Unit of Vaccinia. J Exp Med 74: 263–281. 19871134 10.1084/jem.74.3.263 PMC2135184

[10] Fulginiti VA, Papier A, Lane JM, Neff JM, Henderson DA (2003) Smallpox vaccination: a review, part I. Background, vaccination technique, normal vaccination and revaccination, and expected normal reactions. Clin Infect Dis 37: 241–250. 12856217 10.1086/375824

[11] Belyakov IM, Earl P, Dzutsev A, Kuznetsov VA, Lemon M, et al (2003) Shared modes of protection against poxvirus infection by attenuated and conventional smallpox vaccine viruses. Proc Natl Acad Sci U S A 100: 9458–9463. 12869693 10.1073/pnas.1233578100 PMC170940

[12] Weltzin R, Liu J, Pugachev KV, Myers GA, Coughlin B, et al (2003) Clonal vaccinia virus grown in cell culture as a new smallpox vaccine. Nat Med 9: 1125–1130. 12925845 10.1038/nm916

[13] Qin L, Upton C, Hazes B, Evans DH (2011) Genomic analysis of the vaccinia virus strain variants found in Dryvax vaccine. J Virol 85: 13049–13060. 21976639 10.1128/JVI.05779-11 PMC3233142

[14] Osborne JD, da Silva M, Frace AM, Sammons SA, Olsen-Rasmussen M, et al (2007) Genomic differences of Vaccinia virus clones from Dryvax smallpox vaccine: the Dryvax-like ACAM2000 and the mouse neurovirulent Clone-3. Vaccine 25: 8807–8832. 18037545 10.1016/j.vaccine.2007.10.040

[15] Garcel A, Perino J, Crance JM, Drillien R, Garin D, et al (2009) Phenotypic and genetic diversity of the traditional Lister smallpox vaccine. Vaccine 27: 708–717. 19059294 10.1016/j.vaccine.2008.11.063

[16] Morikawa S, Sakiyama T, Hasegawa H, Saijo M, Maeda A, et al (2005) An attenuated LC16m8 smallpox vaccine: analysis of full-genome sequence and induction of immune protection. J Virol 79: 11873–11891. 16140764 10.1128/JVI.79.18.11873-11891.2005 PMC1212643

[17] Dong S (2009) Qi Changqing, the founder of vaccinia virus Tiantan strain. Weishengwuxue Mianyixue Jinzhan 37: 1–3.

[18] Lu B, Yu W, Huang X, Wang H, Liu L, et al (2011) Mucosal immunization induces a higher level of lasting neutralizing antibody response in mice by a replication-competent smallpox vaccine: vaccinia Tiantan strain. J Biomed Biotechnol 2011: 970424. 21765641 10.1155/2011/970424 PMC3134386

[19] Kai Zhao, Yihao Zhang, Hemin Li (2007) Medical Biologicology. Beijing: people's Health Publishing House 963–972 p.

[20] Upton C, Slack S, Hunter AL, Ehlers A, Roper RL (2003) Poxvirus orthologous clusters: toward defining the minimum essential poxvirus genome. J Virol 77: 7590–7600. 12805459 10.1128/JVI.77.13.7590-7600.2003 PMC164831

[21] Liu L, Hao Y, Luo Z, Huang Y, Hu X, et al (2012) Broad HIV-1 neutralizing antibody response induced by heterologous gp140/gp145 DNA prime-vaccinia boost immunization. Vaccine 30: 4135–4143. 22561314 10.1016/j.vaccine.2012.04.075 PMC3422682

[22] Abramoff MD, Magalhaes PJ, Ram SJ (2004) Image processing with ImageJ. Biophotonics Int 11: 36–42.

[23] Rice CM, Franke CA, Strauss JH, Hruby DE (1985) Expression of Sindbis virus structural proteins via recombinant vaccinia virus: synthesis, processing, and incorporation into mature Sindbis virions. J Virol 56: 227–239. 4032536 10.1128/jvi.56.1.227-239.1985 PMC252510

[24] Li R, Yu C, Li Y, Lam TW, Yiu SM, et al (2009) SOAP2: an improved ultrafast tool for short read alignment. Bioinformatics 25: 1966–1967. 19497933 10.1093/bioinformatics/btp336

[25] Tcherepanov V, Ehlers A, Upton C (2006) Genome Annotation Transfer Utility (GATU): rapid annotation of viral genomes using a closely related reference genome. BMC Genomics 7: 150. 16772042 10.1186/1471-2164-7-150 PMC1534038

[26] Altschul SF, Madden TL, Schaffer AA, Zhang J, Zhang Z, et al (1997) Gapped BLAST and PSI-BLAST: a new generation of protein database search programs. Nucleic Acids Res 25: 3389–3402. 9254694 10.1093/nar/25.17.3389 PMC146917

[27] Upton C, Hogg D, Perrin D, Boone M, Harris NL (2000) Viral genome organizer: a system for analyzing complete viral genomes. Virus Res 70: 55–64. 11074125 10.1016/s0168-1702(00)00210-0

[28] Thompson JD, Higgins DG, Gibson TJ (1994) CLUSTAL W: improving the sensitivity of progressive multiple sequence alignment through sequence weighting, position-specific gap penalties and weight matrix choice. Nucleic Acids Res 22: 4673–4680. 7984417 10.1093/nar/22.22.4673 PMC308517

[29] Brodie R, Smith AJ, Roper RL, Tcherepanov V, Upton C (2004) Base-By-Base: single nucleotide-level analysis of whole viral genome alignments. BMC Bioinformatics 5: 96. 15253776 10.1186/1471-2105-5-96 PMC481056

[30] Esteban DJ, da Silva M, Upton C (2005) New bioinformatics tools for viral genome analyses at Viral Bioinformatics–Canada. Pharmacogenomics 6: 271–280. 16013958 10.1517/14622416.6.3.271

[31] Garcel A, Crance JM, Drillien R, Garin D, Favier AL (2007) Genomic sequence of a clonal isolate of the vaccinia virus Lister strain employed for smallpox vaccination in France and its comparison to other orthopoxviruses. J Gen Virol 88: 1906–1916. 17554021 10.1099/vir.0.82708-0

[32] Tamura K, Peterson D, Peterson N, Stecher G, Nei M, et al (2011) MEGA5: Molecular evolutionary genetics analysis using maximum likelihood, evolutionary distance, and maximum parsimony methods. Molecular Biology and Evolution 28: 2731–2739. 21546353 10.1093/molbev/msr121 PMC3203626

[33] Ng A, Tscharke DC, Reading PC, Smith GL (2001) The vaccinia virus A41L protein is a soluble 30 kDa glycoprotein that affects virus virulence. J Gen Virol 82: 2095–2105. 11514718 10.1099/0022-1317-82-9-2095

[34] Isaacs SN, Wolffe EJ, Payne LG, Moss B (1992) Characterization of a vaccinia virus-encoded 42-kilodalton class I membrane glycoprotein component of the extracellular virus envelope. J Virol 66: 7217–7224. 1433514 10.1128/jvi.66.12.7217-7224.1992 PMC240424

[35] Gardner JD, Tscharke DC, Reading PC, Smith GL (2001) Vaccinia virus semaphorin A39R is a 50–55 kDa secreted glycoprotein that affects the outcome of infection in a murine intradermal model. J Gen Virol 82: 2083–2093. 11514717 10.1099/0022-1317-82-9-2083

[36] Henderson DA, Moss B (1999) Smallpox and Vaccinia. Vaccines 3rd edition. Philadelphia: Saunders.

[37] Yu W, Fang Q, Zhu W, Wang H, Tien P, et al (2010) One time intranasal vaccination with a modified vaccinia Tiantan strain MVTT(ZCI) protects animals against pathogenic viral challenge. Vaccine 28: 2088–2096. 20045097 10.1016/j.vaccine.2009.12.038 PMC7127290

[38] Monath TP, Caldwell JR, Mundt W, Fusco J, Johnson CS, et al (2004) ACAM2000 clonal Vero cell culture vaccinia virus (New York City Board of Health strain)–a second-generation smallpox vaccine for biological defense. Int J Infect Dis 8 Suppl 2S31–44. 15491873 10.1016/j.ijid.2004.09.002 PMC7110559

[39] Polak MF, Beunders BJ, van der Werff AR, Sanders EW, van Klaveren J, et al (1963) A comparative study of clinical reactions observed after application of several smallpox vaccines in primary vaccination of young adults. Bull WHO 29: 311–322. 14058225 PMC2554966

[40] Marennikova SS, Chimishkyan KL, Maltseva NN, Shelukhina EM, Fedorov VV (1969) Proceedings of the Symposium on Smallpox, Zagreb. Yugoslav Academy of Sciences and Arts.

[41] The compilation report of “The vaccinia experience exchange conference, Beijing, 1974” The vaccinia experience exchange conference. Beijing: National Vaccine and Serum Institute, 1975.

