# Supplementary figures and images for: Genomic Sequence and Virulence of Clonal Isolates of Vaccinia Virus Tiantan, the Chinese Smallpox Vaccine Strain
Source: PLoS One. 2013 Apr 12;8(4):e60557. doi: 10.1371/journal.pone.0060557 (PMC3625194; doi:10.1371/journal.pone.0060557)

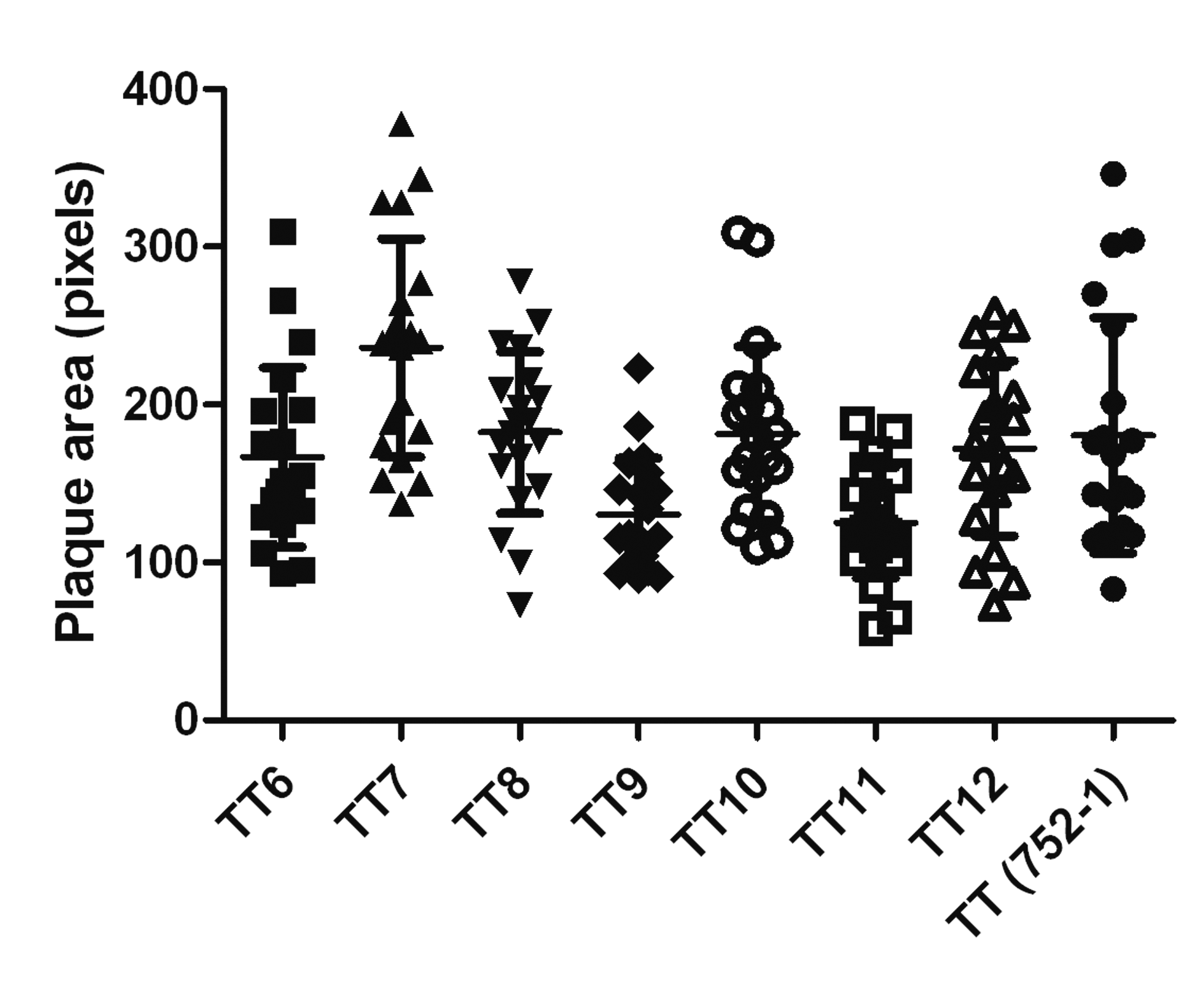

Supplement: Figure S1 — Plaque properties of TT (752-1) and seven TT clones. Each clone of the TT (752-1) was plaque-purified and ∼50 PFU of each clone was plated on a monolayer of CEF cells. The infected cells were cultured for 3 days and then stained with crystal violet and imaged. The plaques were visualized using ImageJ and their sizes were determined. (TIF) [file pone.0060557.s001.tif]
