# Supplementary material for: Genomic Sequence and Virulence of Clonal Isolates of Vaccinia Virus Tiantan, the Chinese Smallpox Vaccine Strain
Source: PLoS One. 2013 Apr 12;8(4):e60557. doi: 10.1371/journal.pone.0060557 (PMC3625194; doi:10.1371/journal.pone.0060557)
Supplement: Table S1 — Identities and GenBank accession numbers of orthopoxviruses cited in this work. (DOC) [file pone.0060557.s002.doc]

| Virus identifier | Virus strain | GenBank Accession no. | Length (bp) |
| --- | --- | --- | --- |
| CMLV-M96 | Camelpox virus M96 | NC_003391 | 205,719 |
| CPXV-BR | Cowpox virus Brighton Red | AF482758 | 224,499 |
| ECTV-Mos | Ectromelia virus Moscow | NC_004105 | 209,771 |
| MPXV-ZAR | Monkeypox virus Zaire-96-I-16 | NC_003310 | 196,858 |
| VARV-BGD75maj | Variola major virus Bangladesh-1975 | L22579 | 186,103 |
| HSPV-MNR76 | Horsepox virus MNR-76 | DQ792504 | 212,633 |
| VACV-Acam2000 | Vaccinia virus Dryvax (Acambis clone 2000) | AY313847 | 199,234 |
| VACV-WR | Vaccinia virus Western Reserve | NC_006998 | 194,711 |
| VACV-DUKE | Vaccinia virus Dryvax Duke (human isolate) | DQ439815 | 199,960 |
| VACV-Lister_VACV107 | Vaccinia virus Lister-V107 | DQ121394 | 189,421 |
| VACV-LC16m8 | Vaccinia virus LC16m8 | AY678275 | 189,158 |
| VACV-Cop | Vaccinia virus Copenhagen | M35027 | 191,738 |
| VACV-MVA | Vaccinia virus Modified Vaccinia Ankara | U94848 | 177,923 |
| VACV-DPP9 | Vaccinia virus Dryvax clone DPP9 | JN654976 | 198,518 |
| VACV-DPP10 | Vaccinia virus Dryvax clone DPP10 | JN654977 | 198,464 |
| VACV-DPP11 | Vaccinia virus Dryvax clone DPP11 | JN654978 | 198,554 |
| VACV-DPP12 | Vaccinia virus Dryvax clone DPP12 | JN654979 | 198,741 |
| VACV-DPP13 | Vaccinia virus Dryvax clone DPP13 | JN654980 | 194,800 |
| VACV-DPP15 | Vaccinia virus Dryvax clone DPP15 | JN654981 | 198,547 |
| VACV-DPP16 | Vaccinia virus Dryvax clone DPP16 | JN654982 | 198,820 |
| VACV-DPP17 | Vaccinia virus Dryvax clone DPP17 | JN654983 | 191,709 |
| VACV-DPP19 | Vaccinia virus Dryvax clone DPP19 | JN654984 | 198,609 |
| VACV-DPP20 | Vaccinia virus Dryvax clone DPP20 | JN654985 | 198,699 |
| VACV-DPP21 | Vaccinia virus Dryvax clone DPP21 | JN654986 | 194,916 |
| VACV-Tian Tan | Vaccinia virus TT (Temple of Heaven) | AF095689.1 | 189,274 |
