# Supplementary material for: Genomic Sequence and Virulence of Clonal Isolates of Vaccinia Virus Tiantan, the Chinese Smallpox Vaccine Strain
Source: PLoS One. 2013 Apr 12;8(4):e60557. doi: 10.1371/journal.pone.0060557 (PMC3625194; doi:10.1371/journal.pone.0060557)
Supplement: Supplementary file 3 [file pone.0060557.s003.doc]

## Sheet: Sheet1

	VACV-TT clones							Reference Genomes
Gene Name	TT8	TT9	TT10	TT11	TT12	Orthologs a	Description	VACV-Cop		VACV-Lister_ VACV107		VACV-Acam 2000		VACV-WR
	gene size (bp)	gene size (bp)	gene size (bp)	gene size (bp)	gene size (bp)			ortholog name	gene size (bp)	ortholog name	gene size (bp)	ortholog name	gene size (bp)	ortholog name	gene size (bp)
VACV_TT_001 (ITR)	720	726	720	735	729	  VACWR001/218, Cop-C23L/B29R	Chemokine-binding protein	Cop-C23L	735	List001	777	VACAC2_001	726	VACWR001	735
VACV_TT_002 (ITR)	204	204	204	204	204	  VV_Cop-C ORF H b	Unknown	VV_Cop-C ORF H	204
VACV_TT_003 (ITR)	438	369	438	360	429	  VACWR004/215, Cop-C22L/B28R	TNF alpha receptor (CrmB)	Cop-C22L	369	List002*	369	VACAC2_003	369	VACWR004	369
VACV_TT_004 (ITR)	360	360	363	453	597	  Cop-C18L/B24R 	Ankyrin-like protein	Cop-C18L	453	List004*A	438	VACAC2_007	597
VACV_TT_005 (ITR)	540	735	1134	549	1140	  Cop-C17L/B23R	Ankyrin-like protein	Cop-C17L	1161	List004*B	1275	VACAC2_008	1149
VACV_TT_005.1 (ITR) c				492		  Cop-C17L/B23R	Ankyrin-like protein
VACV_TT_006 (ITR)	546	546	546	546	546	  Cop-C16L/B22R	Unknown	Cop-C16L	546	List005	444	VACAC2_009	546
VACV_TT_007 (ITR)	210	210	210	210	210	  Cop-C15L/B21R	Unknown	Cop-C15L	276	List006	270	VACAC2_010	270
VACV_TT_008 (ITR)	423	426	423	426	426	  VACWR009/210, Cop-C11R	EGF growth factor	Cop-C11R	429	List009	423	VACAC2_014	420	VACWR009	423
VACV_TT_009 (ITR)	996	996	996	996	996	  VACWR010/209, Cop-C10L	IL-1 receptor antagonist	Cop-C10L	996	List010	996	VACAC2_015	996	VACWR010	996
VACV_TT_010	198	198	198	198	198	  VV_Cop-C ORF E	Unknown	VV_Cop-C ORF E	198
VACV_TT_011	252	252	363	153	252	  VACWR011/208, VARBSH-D6R/005	Zinc finger-like protein, apoptosis, host defense modulator			List011	543	VACAC2_017	252	VACWR011	546
VACV_TT_012	189	189	189	189	189	  VACWR012/207, VARBSH-D6R/005	Zinc finger-like protein, apoptosis, host defense modulator			List012*	201	VACAC2_018	189	VACWR012	189
VACV_TT_013	381	381	381	381	381	  VACWR013, VARBSH-D7L/006	Soluble IL-18  binding protein (Bsh-D7L), host defense modulator			List013	381	VACAC2_019	375	VACWR013	381
VACV_TT_014	273	273	273	273	273	  VACWR014, CPXVBR-V024	Ankyrin-like protein (Bang-D8L), 77 kDa cowpox host-range protein					VACAC2_020	273	VACWR014	714
VACV_TT_015	429	429	429	429	429	  VACWR014, CPXVBR-V024	Ankyrin-like protein (Bang-D8L), 77 kDa cowpox host-range protein			List014*D	429	VACAC2_021	429	VACWR014	714
VACV_TT_016	192	426		414	399	  VACWR015, CPXVBR-V024	Ankyrin-like protein (Bang-D8L), 77 kDa cowpox host-range protein					VACAC2_022	408	VACWR015	414
VACV_TT_017	234	234	234	234	234	  VACWR016, CPXVBR-V024	Ankyrin-like protein (Bang-D8L), 77 kDa cowpox host-range protein			List014*B	234	VACAC2_023	234	VACWR016	234
VACV_TT_018	216	216	216	216	216	  VACWR017, CPXVBR-V024	Ankyrin-like protein (Bang-D8L), 77 kDa cowpox host-range protein					VACAC2_024	216	VACWR017	216
VACV_TT_019	180	180	180	180	180	  VACV-Lister_VACV107-List015	Unknown			List015	180			VACWR018	183
VACV_TT_020	1905	1905	1899	1905	1905	  VACWR019, Cop-C9L	Ankyrin-like protein	Cop-C9L	1905	List016	1905	VACAC2_026	1905	VACWR019	1905
VACV_TT_021	213	213	213	213	213	  VV_Cop-C ORF D	Unknown	VV_Cop-C ORF D	213
VACV_TT_022	534	534	534	534	534	  VACWR020, Cop-C8L	Unknown	Cop-C8L	555	List017	534	VACAC2_027	534	VACWR020	534
VACV_TT_023	249	249	249	249	249	  VV_Cop-C ORF C	Unknown	VV_Cop-C ORF C	249
VACV_TT_024	225	225	225	225	225	  VV_Cop-C ORF B	Unknown	VV_Cop-C ORF B	219
VACV_TT_025	453	453	453	453	453	  VACWR021, Cop-C7L	Host range virulence factor	Cop-C7L	453	List018	453	VACAC2_028	453	VACWR021	453
VACV_TT_026	456	363	456	456	456	  VACWR022, Cop-C6L	Unknown	Cop-C6L	456	List019	456	VACAC2_029	456	VACWR022	456
VACV_TT_027	615	615	561	615	615	  VACWR023, Cop-C5L	Kelch-like protein	Cop-C5L	615	List020	615	VACAC2_030	615	VACWR023	615
VACV_TT_028	951	570	189	594	951	  VACWR024, Cop-C4L	IL-1 receptor antagonist	Cop-C4L	951	List021	951	VACAC2_031	189	VACWR024	951
VACV_TT_028.1 c			387			  VACWR024, Cop-C4L	IL-1 receptor antagonist					VACAC2_032	411
VACV_TT_029	792	786	786	792	792	  VACWR025, Cop-C3L	Secreted complement binding protein	Cop-C3L	792	List022	792	VACAC2_034	792	VACWR025	792
VACV_TT_030	210	210	180	210	180	  VV_Cop-C ORF A	Unknown	VV_Cop-C ORF A	210
VACV_TT_031	1539	1539	1539	1539	1539	  VACWR026, Cop-C2L	Kelch-like protein	Cop-C2L	1539	List023	1539	VACAC2_035	1521	VACWR026	1539
VACV_TT_032	675	675	675	690	675	  VACWR027, Cop-C1L	Unknown	Cop-C1L	675	List024	675	VACAC2_036	675	VACWR027	690
VACV_TT_033	354	354	354	354	354	  VACWR028, Cop-N1L 	Virokine, NF-κB inhibitor	Cop-N1L	354	List025	354	VACAC2_037	354	VACWR028	354
VACV_TT_034	528	528	528	528	528	  VACWR029, Cop-N2L	Alpha amanitin sensitivity protein	Cop-N2L	528	List026	528	VACAC2_038	528	VACWR029	528
VACV_TT_035	1413	1413	1413	1413	1413	  VACWR030, Cop-M1L	Ankyrin-like protein	Cop-M1L	1419	List027	1413	VACAC2_041	1410	VACWR030	1419
VACV_TT_036	663	663	663	663	663	  VACWR031, Cop-M2L	NF-κB inhibitor	Cop-M2L	663	List028	663	VACAC2_042	663	VACWR031	663
VACV_TT_037	855	855	855	855	855	  VACWR032, Cop-K1L	Ankyrin, NF-κB inhibitor	Cop-K1L	855	List029	855	VACAC2_043	855	VACWR032	855
VACV_TT_038	1110	1110	1110	1110	1110	  VACWR033, Cop-K2L	Serpin (serine protease inhibitor) (SPI-3)	Cop-K2L	1110	List030	1110	VACAC2_044	1110	VACWR033	1110
VACV_TT_039	246	246	246	246	246	  VV_Cop-K ORF A	Unknown	VV_Cop-K ORF A	246
VACV_TT_040	240	240	240	240	240	  VV_Cop-K ORF B	Unknown	VV_Cop-K ORF B	240
VACV_TT_041	267	267	267	267	267	  VACWR034, Cop-K3L	IFN resistance, elF2 alpha-like PKR inhibitor	Cop-K3L	267	List031	267	VACAC2_045	267	VACWR034	267
VACV_TT_042	1275	1275	1275	1275	1275	  VACWR035, Cop-K4L	Nicking-joining enzyme	Cop-K4L	1275	Lis032	1275	VACAC2_046	1275	VACWR035	1275
VACV_TT_043	366	366	366	366	366	  VACWR037, Cop-K5L	Monoglyceride lipase	Cop-K5L	411	List033	528	VACAC2_047	411	VACWR037	405
VACV_TT_044	255	246	246	255	255	  VACWR038, Cop-K6L	Monoglyceride lipase	Cop-K6L	246	List034	246	VACAC2_048	246	VACWR038	246
VACV_TT_045	450	450	450	450	450	  VACWR039, Cop-K7R	Unknown	Cop-K7R	450	List035	450	VACAC2_049	450	VACWR039	450
VACV_TT_046	723	681	681	681	681	  VACWR040, Cop-F1L	Apoptosis inhibitor (mitochondrial-associated)	Cop-F1L	681	List036	681	VACAC2_050	681	VACWR040	681
VACV_TT_047	444	444	444	444	444	  VACWR041, Cop-F2L	dUTPase	Cop-F2L	444	List037	444	VACAC2_051	444	VACWR041	444
VACV_TT_048	1443	1443	1443	1443	1443	  VACWR042, Cop-F3L	Kelch-like protein	Cop-F3L	1443	List038	1443	VACAC2_052	1443	VACWR042	1443
VACV_TT_049	960	960	960	960	960	  VACWR043, Cop-F4L 	Ribonucleotide reductase small subunit	Cop-F4L	960	List039	960	VACAC2_053	960	VACWR043	960
VACV_TT_050	237	237	237	237	237	  VV_Cop-F ORF B	Unknown	VV_Cop-F ORF B	237
VACV_TT_051	291	291	291	291	291	  VV_Cop-F ORF C	Unknown	VV_Cop-F ORF C	291
VACV_TT_052	966	969	969	969	969	  VACWR044, Cop-F5L	36kDa major membrane protein	Cop-F5L	966	List040	966	VACAC2_054	966	VACWR044	969
VACV_TT_053	225	225	225	225	225	  VACWR045, Cop-F6L	Unknown	Cop-F6L	225	List041	225	VACAC2_055	225	VACWR045	225
VACV_TT_054	249	249	249	243	249	  VACWR046, Cop-F7L	Unknown	Cop-F7L	279	List042	243	VACAC2_056	243	VACWR046	243
VACV_TT_055	198	198	198	198	198	  VACWR047, Cop-F8L	Cytoplasmic protein	Cop-F8L	198	List043	198	VACAC2_057	198	VACWR047	198
VACV_TT_056	639	639	639	639	639	  VACWR048, Cop-F9L	S-S bond formation pathway protein	Cop-F9L	639	List044	639	VACAC2_058	639	VACWR048	639
VACV_TT_057	1320	1320	1320	1320	1320	  VACWR049, Cop-F10L	Ser/Thr kinase	Cop-F10L	1320	List045	1320	VACAC2_059	1320	VACWR049	1320
VACV_TT_058	258	258	258	258	258	  VV_Cop-F ORF D	Unknown	VV_Cop-F ORF D	258
VACV_TT_059	1065	1065	1065	1065	1065	  VACWR050, Cop-F11L	Unknown	Cop-F11L	1065	List046	1065	VACAC2_060	1047	VACWR050	1047
VACV_TT_060	1908	1908	1908	1908	1908	  VACWR051, Cop-F12L	IEV associated protein	Cop-F12L	1908	List047	1908	VACAC2_061	1908	VACWR051	1908
	VACV-TT clones							Reference Genomes
Gene Name	TT8	TT9	TT10	TT11	TT12	Orthologs a	Description	VACV-Cop		VACV-Lister_ VACV107		VACV-Acam 2000		VACV-WR
	gene size (bp)	gene size (bp)	gene size (bp)	gene size (bp)	gene size (bp)			ortholog name	gene size (bp)	ortholog name	gene size (bp)	ortholog name	gene size (bp)	ortholog name	gene size (bp)
VACV_TT_061	216	216	216	216	216	  VV_Cop-F ORF E	Unknown	VV_Cop-F ORF E	216
VACV_TT_062	1119	1119	1119	1119	1119	  VACWR052, Cop-F13L	EEV phospholipase, major IEV antigen	Cop-F13L	1119	List048	1119	VACAC2_062	1119	VACWR052	1119
VACV_TT_063	222	222	222	222	222	  VACWR053, Cop-F14L	Unknown	Cop-F14L	222	List049	222	VACAC2_063	222	VACWR053	222
VACV_TT_063.5	150	150	150	150	150	  VACWR53.5	IMV protein							VACWR053.5	150
VACV_TT_064	225	225		225	225	  VV_Cop-F ORF F	Unknown	VV_Cop-F ORF F	225
VACV_TT_065	477	477	477	477	477	  VACWR054, Cop-F15L	Unknown	Cop-F15L	477	List050	477	VACAC2_064	477	VACWR054	444
VACV_TT_066	696	696	696	696	696	  VACWR055, Cop-F16L	Unknown	Cop-F16L	696	List051	696	VACAC2_065	696	VACWR055	696
VACV_TT_067	306	306	306	306	306	  VACWR056, Cop-F17R	DNA-binding phosphoprotein, VP11	Cop-F17R	306	List052	306	VACAC2_066	306	VACWR056	306
VACV_TT_068	213	213	213	213	213	  VV_Cop-E ORF A	Unknown	VV_Cop-E ORF A	213
VACV_TT_069	1440	1440	1440	1440	1440	  VACWR057, Cop-E1L	Poly-A polymerase large subunit (VP55)	Cop-E1L	1440	List053	1440	VACAC2_067	1440	VACWR057	1440
VACV_TT_070	2214	2214	2214	2214	2214	  VACWR058, Cop-E2L	Unknown	Cop-E2L	2214	List054	2214	VACAC2_068	2214	VACWR058	2214
VACV_TT_071	573	573	573	573	573	  VACWR059, Cop-E3L	IFN resistance, PKR inhibitor	Cop-E3L	573	List055	573	VACAC2_069	573	VACWR059	573
VACV_TT_072	780	780	780	780	780	  VACWR060, Cop-E4L	RNA polymerase (RPO30)	Cop-E4L	780	List056	780	VACAC2_070	780	VACWR060	780
VACV_TT_073	1026	1026	1026	1026	1026	  VACWR061, Cop-E5R	Virosome component	Cop-E5R	996	List057	996	VACAC2_071	570	VACWR061	1026
VACV_TT_074	321	321	321	321	321	  VV_Cop-E ORF B	Unknown	VV_Cop-E ORF B	321
VACV_TT_075	1704	1704	1704	1704	1704	  VACWR062, Cop-E6R	Unknown	Cop-E6R	1704	List058	1704	VACAC2_073	1704	VACWR062	1704
VACV_TT_076	501	501	501	501	501	  VACWR063, Cop-E7R	Soluble/myristyl EEV	Cop-E7R	501	List059	501	VACAC2_074	501	VACWR063	501
VACV_TT_077	213	213	213	213	213	  VV_Cop-E ORF C	Unknown	VV_Cop-E ORF C	213
VACV_TT_078	822	822	822	822	822	  VACWR064, Cop-E8R	ER-localized MP	Cop-E8R	822	List060	822	VACAC2_075	822	VACWR064	822
VACV_TT_079	201	201	201	201	201	  VV_Cop-E ORF D	Unknown	VV_Cop-E ORF D	201
VACV_TT_080	3021	3021	3021	3021	3021	  VACWR065, Cop-E9L	DNA polymerase	Cop-E9L	3021	List061	3021	VACAC2_076	3018	VACWR065	3021
VACV_TT_081	198	198	198	198	198	  VV_Cop-E ORF E	Unknown	VV_Cop-E ORF E	198
VACV_TT_082	288	288	288	288	288	  VACWR066, Cop-E10R	S-S bond formation pathway protein, Sulfhydryl oxidase	Cop-E10R	288	List062	288	VACAC2_077	288	VACWR066	288
VACV_TT_083	390	390	390	390	390	  VACWR067, Cop-E11L	Virion core protein	Cop-E11L	390	List063	390	VACAC2_078	390	VACWR067	390
VACV_TT_084	2001	2001	2001	2001	2001	  VACWR068, Cop-O1L	Unknown	Cop-O1L	2001	List064	2001	VACAC2_079	2001	VACWR068	2001
VACV_TT_085	348	348	348	348	348	  VV_Cop-E ORF F	Unknown	VV_Cop-E ORF F	348
VACV_TT_086	189	189	189	189	189	  CPV-B-078A (CPV-B)	Unknown
VACV_TT_087	327	327	327	327	327	  VACWR069, Cop-O2L	Glutaredoxin 1	Cop-O2L	327	List065	327	VACAC2_080	327	VACWR069	327
VACV_TT_088	939	939	939	939	939	  VACWR070, Cop-I1L	DNA-binding core protein	Cop-I1L	939	List066	939	VACAC2_081	939	VACWR070	939
VACV_TT_089	222	222	222	222	222	  VACWR071, Cop-I2L	Unknown	Cop-I2L	222	List067	222	VACAC2_082	222	VACWR071	222
VACV_TT_090	810	810	810	810	810	  VACWR072, Cop-I3L	ssDNA-binding phosphoprotein	Cop-I3L	810	List068	810	VACAC2_083	810	VACWR072	810
VACV_TT_091	2316	2316	2316	2316	2316	  VACWR073, Cop-I4L	Ribonucleotide reductase large subunit	Cop-I4L	2316	List069	2316	VACAC2_084	2316	VACWR073	2316
VACV_TT_092	234	234	234	234	234	  VV_Cop-I ORF A	Unknown	VV_Cop-I ORF A	234
VACV_TT_093	240	240	240	240	240	  VACWR074, Cop-I5L	IMV protein VP13	Cop-I5L	240	List070	240	VACAC2_085	240	VACWR074	240
VACV_TT_094	1149	1149	1149	1149	1149	  VACWR075, Cop-I6L	Telomere-binding protein	Cop-I6L	1149	List071	1149	VACAC2_086	1149	VACWR075	1149
VACV_TT_095	1272	1272	1272	1272	1272	  VACWR076, Cop-I7L	Virion core protease	Cop-I7L	1272	List072	1272	VACAC2_087	1272	VACWR076	1272
VACV_TT_096	2031	2031	2031	2031	2031	  VACWR077, Cop-I8R	RNA-helicase, NPH-II	Cop-I8R	2031	List073	2031	VACAC2_088	2031	VACWR077	2031
VACV_TT_097	1776	1776	1776	1776	1776	  VACWR078, Cop-G1L	Metallo protease	Cop-G1L	1776	List074	1776	VACAC2_089	1776	VACWR078	1776
VACV_TT_098	336	336	336	336	336	  VACWR079, Cop-G3L	Unknown	Cop-G3L	336	List075	336	VACAC2_090	336	VACWR079	336
VACV_TT_099	663	663	663	663	663	  VACWR080, Cop-G2R	VLTF (late transcription elongation factor)	Cop-G2R	663	List076	663	VACAC2_091	663	VACWR080	663
VACV_TT_100	375	375	375	375	375	  VACWR081, Cop-G4L	Glutaredoxin 2	Cop-G4L	375	List077	375	VACAC2_092	375	VACWR081	375
VACV_TT_101	1305	1305	1305	1305	1305	  VACWR082, Cop-G5R	FEN1-like nuclease	Cop-G5R	1305	List078	1305	VACAC2_093	1305	VACWR082	1305
VACV_TT_101.5	192	192	192	192	192	  VACWR083, Cop-G5.5R	RNA polymerase (RPO7)	Cop-G5.5R	192	List079	192	VACAC2_094	192	VACWR083	192
VACV_TT_102	498	498	498	498	498	  VACWR084, Cop-G6R	Virulence factor, NIpC/P60 superfamily protein	Cop-G6R	498	List080	498	VACAC2_095	498	VACWR084	498
VACV_TT_103	1116	1116	1116	1116	1116	  VACWR085, Cop-G7L	Virion assembly protein 	Cop-G7L	1116	List081	1116	VACAC2_096	1116	VACWR085	1116
VACV_TT_104	399	399	399	399	399	  VV_Cop-G ORF A	Unknown	VV_Cop-G ORF A	399
VACV_TT_105	219	219	219	219	219	  VV_Cop-G ORF B	Unknown	VV_Cop-G ORF B	219
VACV_TT_106	783	783	783	783	783	  VACWR086, Cop-G8R	VLTF-1 (Viral late gene transcription factor)	Cop-G8R	783	List082	783	VACAC2_097	783	VACWR086	783
VACV_TT_107	1023	1023	1023	1023	1023	  VACWR087, Cop-G9R	Entry-fusion complex protein	Cop-G9R	1023	List083	1023	VACAC2_098	1023	VACWR087	1023
VACV_TT_108	753	753	753	753	753	  VACWR088, Cop-L1R	Myristylated MP IMV	Cop-L1R	753	List084	753	VACAC2_099	753	VACWR088	753
VACV_TT_109	258	258	258	264	258	  VACWR089, Cop-L2R	Unknown	Cop-L2R	264	List085	264	VACAC2_100	258	VACWR089	264
VACV_TT_110	1053	1053	1053	1053	1053	  VACWR090, Cop-L3L	Internal virion protein	Cop-L3L	1053	List086	1053	VACAC2_101	1053	VACWR090	1053
VACV_TT_111	756	756	756	756	756	  VACWR091, Cop-L4R	ssDNA/ssRNA binding protein	Cop-L4R	756	List087	756	VACAC2_102	756	VACWR091	756
VACV_TT_112	387	387	387	387	387	  VACWR092, Cop-L5R	Entry and fusion IMV protein	Cop-L5R	387	List088	387	VACAC2_103	387	VACWR092	387
VACV_TT_113	462	462	462	462	462	  VACWR093, Cop-J1R	Virion morphogenesis protein	Cop-J1R	462	List089	462	VACAC2_104	462	VACWR093	462
VACV_TT_114	534	534	534	534	534	  VACWR094, Cop-J2R	Thymidine kinase	Cop-J2R	534	List090	534	VACAC2_105	534	VACWR094	534
VACV_TT_115	1002	1002	1002	1002	1002	  VACWR095, Cop-J3R	Multifunctional poly-A polymerase-small subunit (VP39), Cap methyltransferase	Cop-J3R	1002	List091	1002	VACAC2_106	1002	VACWR095	1002
VACV_TT_116	558	558	558	558	558	  VACWR096, Cop-J4R	RNA polymerase (RPO22)	Cop-J4R	558	List092	558	VACAC2_107	558	VACWR096	558
VACV_TT_117	402	402	402	402	402	  VACWR097, Cop-J5L	Late 16kDa Cop-membrane protein	Cop-J5L	402	List093	402	VACAC2_108	402	VACWR097	402
VACV_TT_118	3861	3861	3861	3861	3861	  VACWR098, Cop-J6R	RNA polymerase (RPO147)	Cop-J6R	3861	List094	3861	VACAC2_109	3861	VACWR098	3861
VACV_TT_119	216	216	216	216	216	  VV_Cop-G ORF B	Unknown	VV_Cop-H ORF A	216
VACV_TT_120	516	516	516	516	516	  VACWR099, Cop-H1L	Tyr/Ser protein phosphatase	Cop-H1L	516	List095	516	VACAC2_110	516	VACWR099	516
	VACV-TT clones							Reference Genomes
Gene Name	TT8	TT9	TT10	TT11	TT12	Orthologs a	Description	VACV-Cop		VACV-Lister_ VACV107		VACV-Acam 2000		VACV-WR
	gene size (bp)	gene size (bp)	gene size (bp)	gene size (bp)	gene size (bp)			ortholog name	gene size (bp)	ortholog name	gene size (bp)	ortholog name	gene size (bp)	ortholog name	gene size (bp)
VACV_TT_121	570	570	570	570	570	  VACWR100, Cop-H2R	Component of multiprotein entry-fusion complex	Cop-H2R	570	List096	570	VACAC2_111	570	VACWR100	570
VACV_TT_122	975	975	975	975	975	  VACWR101, Cop-H3L	IMV heparin binding surface protein	Cop-H3L	975	List097	975	VACAC2_112	975	VACWR101	975
VACV_TT_123	2388	2388	2388	2388	2388	  VACWR102, Cop-H4L	RAP94 (RNA polymerasel associated protein)	Cop-H4L	2388	List098	2388	VACAC2_113	2388	VACWR102	2388
VACV_TT_124	612	612	612	612	612	  VACWR103, Cop-H5R	VLTF-4 (Viral late transcription factor-4)	Cop-H5R	612	List099	612	VACAC2_114	612	VACWR103	612
VACV_TT_125	945	945	945	945	945	  VACWR104, Cop-H6R	Topoisomerase type I	Cop-H6R	945	List100	945	VACAC2_115	945	VACWR104	945
VACV_TT_126	183	183	183	183	183	  CPV-B-116 (CPV-B)	Unknown
VACV_TT_127	441	441	441	441	441	  VACWR105, Cop-H7R	Unknown	Cop-H7R	441	List101	441	VACAC2_116	441	VACWR105	441
VACV_TT_128	2535	2535	2535	2535	2535	  VACWR106, Cop-D1R	mRNA capping enzyme large subunit, VTF (Viral transcription termination factor )	Cop-D1R	2535	List102	2535	VACAC2_117	2535	VACWR106	2535
VACV_TT_129	255	255	255	255	255	  VV_Cop-D ORF A	Unknown	VV_Cop-D ORF A	255
VACV_TT_130	441	441	441	441	441	  VACWR107, Cop-D2L	Virion core protein	Cop-D2L	441	List103	441	VACAC2_118	441	VACWR107	441
VACV_TT_131	243	243	243	243	243	  VV_Cop-D ORF B	Unknown	VV_Cop-D ORF B	243
VACV_TT_132	714	714	714	714	714	  VACWR108, Cop-D3R	Virion core protein	Cop-D3R	714	List104	714	VACAC2_119	714	VACWR108	714
VACV_TT_133	657	657	657	657	657	  VACWR109, Cop-D4R	Uracil-DNA glycosylase	Cop-D4R	657	List105	657	VACAC2_120	657	VACWR109	657
VACV_TT_134	210	210	210	210	210	  VV_Cop-D ORF C	Unknown 	VV_Cop-D ORF C	210
VACV_TT_135	2358	2358	2358	2358	2358	  VACWR110, Cop-D5R	DNA-independent NTPase	Cop-D5R	2358	List106	2358	VACAC2_121	2358	VACWR110	2358
VACV_TT_136	219	219	219	219	219	  VV_Cop-D ORF D	Unknown	VV_Cop-D ORF D	219
VACV_TT_137	243	243	243	243	243	  VV_Cop-D ORF E	Unknown	VV_Cop-D ORF E	243
VACV_TT_138	1914	1914	1914	1914	1914	  VACWR111, Cop-D6R	VETF (Viral early transcription factor small subunit )	Cop-D6R	1914	List107	1914	VACAC2_122	1914	VACWR111	1914
VACV_TT_139	486	486	486	486	486	  VACWR112, Cop-D7R	RNA polymerase (RPO18)	Cop-D7R	486	List108	486	VACAC2_123	486	VACWR112	486
VACV_TT_140	915	915	915	915	915	  VACWR113, Cop-D8L	Carbonic anhydrase, IMV membrane protein	Cop-D8L	915	List109	915	VACAC2_124	915	VACWR113	915
VACV_TT_141	642	642	642	642	642	  VACWR114, Cop-D9R	NTP-phosphohydrolase (NTP-PPH) contains mutT-like motif	Cop-D9R	642	List110	642	VACAC2_125	642	VACWR114	642
VACV_TT_142	747	747	747	747	747	  VACWR115, Cop-D10R	NPH-PPH RNA levels regulator contains mutT-like motif 	Cop-D10R	747	List111	747	VACAC2_126	747	VACWR115	747
VACV_TT_143	1896	1896	1896	1896	1896	  VACWR116, Cop-D11L	ATPase, NPH-I (nucleoside triphosphate phosphohydrolase-I), Helicase, Virion	Cop-D11L	1896	List112	1896	VACAC2_127	1896	VACWR116	1896
VACV_TT_144	210	210	210	210	210	  VV_Cop-D ORF F	Unknown	VV_Cop-D ORF F	210
VACV_TT_145	273	273	273	273	273	  VV_Cop-D ORF G	Unknown	VV_Cop-D ORF G	273
VACV_TT_146	864	864	864	864	864	  VACWR117, Cop-D12L	mRNA capping enzyme small subunit, VTF (transcription termination factor) 	Cop-D12L	864	List113	864	VACAC2_128	864	VACWR117	864
VACV_TT_147	219	219	219	219	219	  VACV-Tian_Tan-134	Unknown
VACV_TT_148	1656	1656	1656	1656	1656	  VACWR118, Cop-D13L	Trimeric virion coat protein (rifampicin resistance)	Cop-D13L	1656	List114	1656	VACAC2_129	1656	VACWR118	1656
VACV_TT_149	225	225	225	225	225	  VV_Cop-D ORF I	Unknown	VV_Cop-D ORF I	225
VACV_TT_150	453	453	453	453	453	  VACWR119, Cop-A1L	VLTF-2 (viral late transcription factor-2)	Cop-A1L	453	List115	453	VACAC2_130	453	VACWR119	453
VACV_TT_151	675	675	675	675	675	  VACWR120, Cop-A2L	VLTF-3 (viral late transcription factor-3)	Cop-A2L	675	List116	675	VACAC2_131	675	VACWR120	675
VACV_TT_151.5	231	231	231	231	231	  VACWR121, Cop-A2.5L	Thioredoxin-like protein, S-S bond formation pathway protein	Cop-A2.5L	231	List117	231	VACAC2_132	231	VACWR121	231
VACV_TT_152	1935	1935	1935	1935	1935	  VACWR122, Cop-A3L	P4b (precursor of core protein 4b)	Cop-A3L	1935	List118	1935	VACAC2_133	1935	VACWR122	1935
VACV_TT_153	360	360	360	360	360	  VV_Cop-A ORF A	Unknown	VV_Cop-A ORF A	360
VACV_TT_154	852	852	852	846	852	  VACWR123, Cop-A4L	39kDa core protein	Cop-A4L	846	List119	846	VACAC2_134	846	VACWR123	846
VACV_TT_155	261	261	261	261	261	  VV_Cop-A ORF B	Unknown	VV_Cop-A ORF B	261
VACV_TT_156	495	495	495	495	495	  VACWR124, Cop-A5R	RNA polymerase (RPO19)	Cop-A5R	495	List120	495	VACAC2_135	495	VACWR124	495
VACV_TT_157	1119	1119	1119	1119	1119	  VACWR125, Cop-A6L	Virion morphogenesis, Virion core protein	Cop-A6L	1119	List121	1119	VACAC2_136	1119	VACWR125	1119
VACV_TT_158	2133	2133	2133	2133	2133	  VACWR126, Cop-A7L	VETF-L (Viral early gene transcription factor large subunit)	Cop-A7L	2133	List122	2133	VACAC2_137	2133	VACWR126	2133
VACV_TT_159	387	387	387	387	387	  VV_Cop-A ORF C	Unknown	VV_Cop-A ORF C	387
VACV_TT_160	267	267	267	267	267	  VV_Cop-A ORF D	Unknown	VV_Cop-A ORF D	267
VACV_TT_161	867	867	867	867	867	  VACWR127, Cop-A8R	VITF-3 (Viral intermediate transcription factor-3)	Cop-A8R	867	List123	867	VACAC2_138	867	VACWR127	867
VACV_TT_162	300	327	312	327	327	  VACWR128, Cop-A9L	IMV membrane protein	Cop-A9L	300	List124	300	VACAC2_139	327	VACWR128	327
VACV_TT_163	2679	2679	2679	2679	2679	  VACWR129, Cop-A10L	P4a (precursor of core protein 4a)	Cop-A10L	2676	List125	2676	VACAC2_140	2676	VACWR129	2676
VACV_TT_164	501	501	501	501	501	  VV_Cop-A ORF E	Unknown	VV_Cop-A ORF E	501
VACV_TT_165	231	231	231	204	231	  VV_Cop-A ORF F	Unknown	VV_Cop-A ORF F	228
VACV_TT_166	228	228	228	228	228	  VV_Cop-A ORF G	Unknown	VV_Cop-A ORF G	228
VACV_TT_167	957	957	957	957	957	  VACWR130, Cop-A11R	Viral membrane formation protein	Cop-A11R	957	List126	957	VACAC2_141	957	VACWR130	957
VACV_TT_168	576	576	579	576	576	  VACWR131, Cop-A12L	Structural protein	Cop-A12L	579	List127	579	VACAC2_142	579	VACWR131	579
VACV_TT_169	213	213	213	213	213	  VACWR132, Cop-A13L	Virion maturation protein	Cop-A13L	213	List128	213	VACAC2_143	213	VACWR132	213
VACV_TT_170	273	273	273	273	273	  VACWR133, Cop-A14L	IMV PO4 MP (phosphorylated IMV membrane protein required for morphogenesis)	Cop-A14L	273	List129	273	VACAC2_144	273	VACWR133	273
VACV_TT_170.5	162	162	162	162	162	  VACWR134, Cop-A14.5L	IMV membrane protein, Virulence factor	Cop-A14.5L	162	List130	162	VACAC2_145	162	VACWR134	162
VACV_TT_171	285	285	285	285	285	  VACWR135, Cop-A15L	Unknown	Cop-A15L	285	List131	285	VACAC2_146	285	VACWR135	285
VACV_TT_172	1137	1137	1137	1137	1137	  VACWR136, Cop-A16L	Soluble myristylprotein, Cell-cell fusion protein	Cop-A16L	1137	List132	1134	VACAC2_147	1137	VACWR136	1134
VACV_TT_173	612	612	612	612	612	  VACWR137, Cop-A17L	IMV MP PO4 (phosphorylated IMV membrane protein required for morphogenesis)	Cop-A17L	612	List133	612	VACAC2_148	612	VACWR137	612
VACV_TT_174	1482	1482	1482	1482	1482	  VACWR138, Cop-A18R	DNA helicase, Transcript release factor	Cop-A18R	1482	List134	1482	VACAC2_149	1482	VACWR138	1482
VACV_TT_175	234	234	234	234	234	  VACWR139, Cop-A19L	Unknown	Cop-A19L	234	List135	234	VACAC2_150	234	VACWR139	234
VACV_TT_176	354	354	354	354	354	  VACWR140, Cop-A21L	Entry and cell to cell fusion protein	Cop-A21L	354	List136	354	VACAC2_151	354	VACWR140	354
VACV_TT_177	1281	1281	1281	1281	1281	  VACWR141, Cop-A20R	DNA processivity factor	Cop-A20R	1281	List137	1281	VACAC2_152	1281	VACWR141	1281
	VACV-TT clones							Reference Genomes
Gene Name	TT8	TT9	TT10	TT11	TT12	Orthologs a	Description	VACV-Cop		VACV-Lister_ VACV107		VACV-Acam 2000		VACV-WR
	gene size (bp)	gene size (bp)	gene size (bp)	gene size (bp)	gene size (bp)			ortholog name	gene size (bp)	ortholog name	gene size (bp)	ortholog name	gene size (bp)	ortholog name	gene size (bp)
VACV_TT_178	387	387	387	387	387	  VV_Cop-A ORF H	Unknown	VV_Cop-A ORF H	387
VACV_TT_179	222	222	222	222	222	  VV_Cop-A ORF I	Unknown	VV_Cop-A ORF I	222
VACV_TT_180	564	564	564	531	564	  VACWR142, Cop-A22R	Holliday junction resolvase	Cop-A22R	531	List138	564	VACAC2_153	564	VACWR142	564
VACV_TT_181	1149	1149	1149	1149	1149	  VACWR143, Cop-A23R	VITF-3 (viral intermediate transcription factor-3) 45kDa subunit	Cop-A23R	1149	List139	1149	VACAC2_154	1149	VACWR143	1149
VACV_TT_182	3495	3495	3495	3495	3495	  VACWR144, Cop-A24R	RNA polymerase132 (RPO132)	Cop-A24R	3495	List140	3495	VACAC2_155	3495	VACWR144	3495
VACV_TT_183	222	222	222	222	222	  VV_Cop-A ORF J	Unknown	VV_Cop-A ORF J	222
VACV_TT_184	639		720	639	720	  VACWR146	Cowpox A-type inclusion protein					VACAC2_157	465	VACWR146	465
VACV_TT_185	207	207		207	207	  VACV-Tian_Tan-171	Unknown
VACV_TT_186	162	162	162	162	162	  MPXV_USA2003_044_143	Cowpox A-type inclusion protein
VACV_TT_187	693	693	684	684	693	  VACWR147	Cowpox A-type inclusion protein					VACAC2_158	684	VACWR147	684
VACV_TT_188	186	186	186	186	186	  VACV-Tian_Tan-173	Unknown
VACV_TT_189	2178	2178	2178	2178	2178	  VACWR148	Cowpox A-type inclusion protein					VACAC2_159	2166	VACWR148	2178
VACV_TT_190	183	183	183	183	183	  VACV-Tian_Tan-175	Unknown
VACV_TT_191	1509	1509	1509	846	1509	  VACWR149, Cop-A26L	P4c (precursor of core protein 4c)	Cop-A26L	969	List142	1509	VACAC2_160	1503	VACWR149	1503
VACV_TT_191.1 c				609		  VACWR149, Cop-A26L	P4c (precursor of core protein 4c)
VACV_TT_192	333	333	333	333	333	  VACWR150, Cop-A27L	IMV surface protein (IMV-cell attachment, fusion, and microtubule transport)	Cop-A27L	333	List143	333	VACAC2_161	333	VACWR150	333
VACV_TT_193	441	441	441	441	441	  VACWR151, Cop-A28L	IMV membrane protein, Virus entry	Cop-A28L	441	List144	441	VACAC2_162	441	VACWR151	441
VACV_TT_194	918	918	918	918	918	  VACWR152, Cop-A29L	RNA polymerase35 (RPO35)	Cop-A29L	918	List145	918	VACAC2_163	918	VACWR152	918
VACV_TT_195	213	213	213	213	213	  VV_Cop-A ORF K	Unknown	VV_Cop-A ORF K	213
VACV_TT_196	234	234	234	234	234	  VACWR153, Cop-A30L	Virion morphogenesis protein 	Cop-A30L	234	List146	234	VACAC2_164	234	VACWR153	234
VACV_TT_197	375	375	375	375	375	  VACWR154, Cop-A31R	Unknown	Cop-A31R	375	List147	375	VACAC2_165	375	VACWR154	375
VACV_TT_198	903	813	813	813	813	  VACWR155, Cop-A32L	ATPase, DNA packaging protein	Cop-A32L	903	List148	903	VACAC2_166	813	VACWR155	813
VACV_TT_199	267	267	267	267	267	  VV_Cop-A ORF L	Unknown	VV_Cop-A ORF L	267
VACV_TT_200	558	558	558	558	558	  VACWR156, Cop-A33R	EEV Glycoprotein 	Cop-A33R	558	List149	558	VACAC2_167	558	VACWR156	558
VACV_TT_201	507	507	507	507	507	  VACWR157, Cop-A34R	C-type lectin-like EEV protein	Cop-A34R	507	List150	507	VACAC2_168	507	VACWR157	507
VACV_TT_202	231	231	231	231	231	  VV_Cop-A ORF M	Unknown	VV_Cop-A ORF M	231
VACV_TT_203	531	531	531	531	531	  VACWR158, Cop-A35R	Unknown	Cop-A35R	531	List151	531	VACAC2_169	531	VACWR158	531
VACV_TT_204	666	666	666	666	666	  VACWR159, Cop-A36R	IEV transmembrane phosphoprotein	Cop-A36R	666	List152	666	VACAC2_170	666	VACWR159	666
VACV_TT_205	792	792	792	792	792	  VACWR160, Cop-A37R	Unknown	Cop-A37R	792	List153	792	VACAC2_171	792	VACWR160	792
VACV_TT_206	252	252	252	252	252	  VV_Cop-A ORF O	Unknown	VV_Cop-A ORF O	252
VACV_TT_207	195	195	195	195	195	  VACWR161, VARGAR-A43R	Unknown			List154	189	VACAC2_172	189	VACWR161	189
VACV_TT_208	183	183	183	183	183	  VACV-REC_GLV_1h68-225	Unknown
VACV_TT_209	834	834	834	834	834	  VACWR162, Cop-A38L	CD47-like protein	Cop-A38L	834	List155	834	VACAC2_173	834	VACWR162	834
VACV_TT_210	1212	687	687	774	774	  VACWR163, Cop-A39R	Semaphorin	Cop-A39R	1212	List156	1212	VACAC2_174	399	VACWR163	888
VACV_TT_210.1 c		429	468	468	468	  VACWR164	Semaphorin					VACAC2_175	429	VACWR164	429
VACV_TT_211	270	270	270	270	270	  VV_Cop-A ORF P	Unknown	VV_Cop-A ORF P	270
VACV_TT_212	480	480	480	480	480	  VACWR165, Cop-A40R	C-type lectin-like type-II membrane protein	Cop-A40R	507	List157	480	VACAC2_176	507	VACWR165	480
VACV_TT_213	660	660	660	660	660	  VACWR166, Cop-A41L 	Secreted virulence factor	Cop-A41L	660	List158	660	VACAC2_177	660	VACWR166	660
VACV_TT_214	402	402	402	402	402	  VACWR167, Cop-A42R	Profilin-like protein, ATI-localized	Cop-A42R	402	List159	402	VACAC2_178	402	VACWR167	402
VACV_TT_215	585	585	585	585	585	  VACWR168, Cop-A43R	Membrane glycoprotein-class I	Cop-A43R	585	List160	585	VACAC2_179	585	VACWR168	585
VACV_TT_216	237	237	237	237	237	  VACWR169 	Unknown			List161	237			VACWR169	237
VACV_TT_217	1041	1041	1041	1041	1041	  VACWR170, Cop-A44L	Hydroxysteroid dehydrogenase	Cop-A44L	1041	List162	1041	VACAC2_181	1041	VACWR170	1041
VACV_TT_218	378	378	378	378	378	  VACWR171, Cop-A45R	Superoxide dismutase-like protein	Cop-A45R	378	List163	378	VACAC2_182	378	VACWR171	378
VACV_TT_219	723	723	723	723	723	  VACWR172, Cop-A46R	IL-1 signaling inhibitor	Cop-A46R	645	List164	723	VACAC2_183	723	VACWR172	723
VACV_TT_220	318	318	318	318	318	  VV_Cop-A ORF Q	Unknown	VV_Cop-A ORF Q	234
VACV_TT_221	759	759	759	759	735	  VACWR173, Cop-A47L	Unknown	Cop-A47L	735	List165	759	VACAC2_184	735	VACWR173	759
VACV_TT_222	684	684	684	684	684	  VACWR174, Cop-A48R	Thymidylate kinase	Cop-A48R	615	List166	684	VACAC2_185	615	VACWR174	684
VACV_TT_223	489	489	489	489	489	  VACWR175, Cop-A49R	Putative phosphotransferase, Anion transport protein 	Cop-A49R	489	List167	489	VACAC2_186	489	VACWR175	489
VACV_TT_224	1659	1659	1659	1659	1659	  VACWR176, Cop-A50R	ATP-dependent DNA ligase	Cop-A50R	1659	List168	1659	VACAC2_187	1659	VACWR176	1659
VACV_TT_225	201	201	201	201	201	  VV_Cop-A ORF R	Unknown	VV_Cop-A ORF R	201
VACV_TT_226	204	204	204	204	204	  VV_Cop-A ORF S	Unknown	VV_Cop-A ORF S	204
VACV_TT_227	1005	1005	1005	1005	1005	  VACWR177, Cop-A51R	Unknown	Cop-A51R	1005	List169	801	VACAC2_188.1	801	VACWR177	1005
VACV_TT_228	573	573	573	573	573	  VACWR178, Cop-A52R	Intracellular TLR and IL-1 signal inhibitor	Cop-A52R	573	List170	573	VACAC2_189	573	VACWR178	573
VACV_TT_229	273				273	  Cop-A54L	Unknown	Cop-A54L	273	List171*	258
VACV_TT_230	399	399	561	561	399	  VACWR179, Cop-A53R	TNF receptor (CrmC)	Cop-A53R	312	List172	561	VACAC2_190	399	VACWR179	312
VACV_TT_231	180	180	180	180	180	  VACV-Lister_VACV107-173	Unknown			List173	180
VACV_TT_232	1695	1695	1695	1695	1695	  VACWR180, Cop-A55R	Kelch-like protein	Cop-A55R	1695	List174	1695	VACAC2_191	1695	VACWR180	1695
VACV_TT_233	948	948	948	948	948	  VACWR181, Cop-A56R	Hemagglutinin	Cop-A56R	948	List175	942	VACAC2_192	930	VACWR181	945
VACV_TT_234	456	456	456	456	456	  VACWR182, Cop-A57R	Guanylate kinase	Cop-A57R	456	List176	456	VACAC2_193	456	VACWR182	456
VACV_TT_235	903	903	903	903	903	  VACWR183, Cop-B1R	Ser/Thr kinase	Cop-B1R	903	List177	903	VACAC2_194	903	VACWR183	903
VACV_TT_236	324	219	219	219	324	  VV_Cop-B ORF A	Unknown	VV_Cop-B ORF A	324
	VACV-TT clones							Reference Genomes
Gene Name	TT8	TT9	TT10	TT11	TT12	Orthologs a	Description	VACV-Cop		VACV-Lister_ VACV107		VACV-Acam 2000		VACV-WR
	gene size (bp)	gene size (bp)	gene size (bp)	gene size (bp)	gene size (bp)			ortholog name	gene size (bp)	ortholog name	gene size (bp)	ortholog name	gene size (bp)	ortholog name	gene size (bp)
VACV_TT_237	660	660	660	660	660	  VACWR184, Cop-B2R	Schlafen	Cop-B2R	660	List178	660	VACAC2_195	660	VACWR184	660
VACV_TT_238	201		201	201	201	  VV_Cop-B ORF B	Unknown	VV_Cop-B ORF B	201
VACV_TT_239	288	288	288	288	288	  VV_Cop-B ORF C	Unknown	VV_Cop-B ORF C	288
VACV_TT_240	375	375	375	375	375	  VACWR185, Cop-B3R	Schlafen	Cop-B3R	375	List179	375	VACAC2_196	375	VACWR185	504
VACV_TT_241	1677	1677	1677	1677	1677	  VACWR186, Cop-B4R	Ankyrin-like protein	Cop-B4R	1677	List181	1677	VACAC2_197	1677	VACWR186	1677
VACV_TT_242	954	954	954	954	954	  VACWR187, Cop-B5R	Complement control, CD46, EEV	Cop-B5R	954	List182	666	VACAC2_198	954	VACWR187	954
VACV_TT_243	522	522	522	522	522	  VACWR188, Cop-B6R	Unknown	Cop-B6R	522	List183	522	VACAC2_199	522	VACWR188	522
VACV_TT_244	216	216	216	216	216	  VV_Cop-B ORF D	Unknown	VV_Cop-B ORF D	216
VACV_TT_245	549	549	549	549	549	  VACWR189, Cop-B7R	Virulence, ER resident	Cop-B7R	549	List184	549	VACAC2_200	549	VACWR189	549
VACV_TT_246	819	819	819	819	816	  VACWR190,Cop-B8R	Soluble interferon-gamma receptor	Cop-B8R	819	List185	819	VACAC2_201	819	VACWR190	819
VACV_TT_247	174	174	174	174	174	  VACV-REC_GLV_1h68-269	Unknown
VACV_TT_248	234	234	234	234	234	  VACWR191, Cop-B9R	Virulence factor	Cop-B9R	234	List186	234	VACAC2_202	234	VACWR191	234
VACV_TT_249	501	501	501	501	501	  VACWR192, Cop-B10R	Kelch-like protein (CPV-GRI-B9R)	Cop-B10R	501	List187	501	VACAC2_203	501	VACWR192	501
VACV_TT_250	231	231	231	219	219	  VACWR193, Cop-B11R	Unknown	Cop-B11R	267	List188	219	VACAC2_204	219	VACWR193	219
VACV_TT_251	852	852	852	852	852	  VACWR194, Cop-B12R	Ser/Thr kinase	Cop-B12R	852	List189	852	VACAC2_205	852	VACWR194	852
VACV_TT_252	1008	351	1008	1008	1008	  VACWR195, Cop-B13R, Cop-B14R	Serpin 1,2,3	Cop-B13R	351	List190*A	351	VACAC2_206	381	VACWR195	1038
VACV_TT_252.1  c		669				  VACWR195, Cop-B13R, Cop-B14R	Serpin 1,2,3	Cop-B14R	669	List190*B	669	VACAC2_207	669	VACWR195	1038
VACV_TT_253	450	450	450	450	450	  VACWR196, Cop-B15R	Unknown	Cop-B15R	450	List191	450	VACAC2_208	450	VACWR196	450
VACV_TT_254	276	174	174	174	174	  VV_Cop-B ORF E	Unknown	VV_Cop-B ORF E	276
VACV_TT_255	981	981	981	981	981	  VACWR197, Cop-B16R	IL-1 beta receptor	Cop-B16R	873	List192	981	VACAC2_209	981	VACWR197	981
VACV_TT_256	228	228	228	228	228	  VV_Cop-B ORF F	Unknown	VV_Cop-B ORF F	228
VACV_TT_257	1023	1023	1023	1023	1023	  VACWR198, Cop-B17L	Unknown	Cop-B17L	1023	List193	1023	VACAC2_210	1023	VACWR198	1023
VACV_TT_258	1725	1725	1725	1725	1725	  VACWR199, Cop-B18R	Ankyrin-like protein	Cop-B18R	1725	List194*	1242	VACAC2_211	1725	VACWR199	1725
VACV_TT_259	1056	1062	1056	1056	1062	  VACWR200, Cop-B19R	IFN-alpha/beta receptor	Cop-B19R	1062			VACAC2_212	798	VACWR200	1056
VACV_TT_260	1809	1794	441	1794	1842	  Cop-B20R	Ankyrin-like protien	Cop-B20R	384
VACV_TT_261 (ITR)	996	996	996	996	996	  VACWR010/209, Cop-C10L	IL-1 Receptor antagonist					VACAC2_227	996	VACWR209	996
VACV_TT_262 (ITR)	423	426	426	426	426	  VACWR009/210, Cop-C11R	EGF growth factor					VACAC2_228	420	VACWR210	423
VACV_TT_263 (ITR)	210	210	210	210	210	  Cop-C15L/B21R	Unknown	Cop-B21R	276
VACV_TT_264 (ITR)	546	546	546	546	546	  Cop-C16L/B22R	Unknown	Cop-B22R	546	List197	444	VACAC2_233	546
VACV_TT_265 (ITR)	540	735	1134	549	1140	  Cop-C17L/B23R	Ankyrin-like protein	Cop-B23R	1161	List198*A	1275	VACAC2_234	1149
VACV_TT_265.1 (ITR) c				492		  Cop-C17L/B23R	Ankyrin-like protein
VACV_TT_266 (ITR)	360	360	363	453	597	  Cop-C18L/B24R	Ankyrin-like protein	Cop-B24R	453			VACAC2_235	597
VACV_TT_267 (ITR)	438	369	438	360	429	  Cop-C22L/B28R	TNF receptor (CrmB)	Cop-B28R	369	List200*	369	VACAC2_239	369	VACWR215	369
VACV_TT_268 (ITR)	720	726	720	735	729	  VACWR001/218, Cop-C23L/B29R	Chemokine binding protein	Cop-B29R	735	List201	777	VACAC2_241	726	VACWR218	735
VACV_TT_269 (ITR)	204	204	204	204	204	  VV_Cop-B ORF I	Unknown	VV_Cop-B ORF I	204

## Sheet: Sheet2

## Sheet: Sheet3
