# Supplementary material for: Genomic Sequence and Virulence of Clonal Isolates of Vaccinia Virus Tiantan, the Chinese Smallpox Vaccine Strain
Source: PLoS One. 2013 Apr 12;8(4):e60557. doi: 10.1371/journal.pone.0060557 (PMC3625194; doi:10.1371/journal.pone.0060557)
Supplement: Supplementary file 4 [file pone.0060557.s004.doc]

## Sheet: Sheet1

	VACV-TT clones									Reference VACVs
Gene name	TT11	TT8		TT9		TT10		TT12		Cop		ListerV107		Acam 2000		WR
	gene size (bp)	gene size (bp)	similarity vs TT11 (%)	gene size (bp)	similarity vs TT11 (%)	gene size (bp)	similarity vs TT11 (%)	gene size (bp)	similarity vs TT11 (%)	ortholog size (bp)	similarity vs TT11 (%)	ortholog size (bp)	similarity vs TT11 (%)	ortholog size (bp)	similarity vs TT11 (%)	ortholog size (bp)	similarity vs TT11 (%)
VACV_TT_001 (ITR)	735	720	98	726	98.8	720	98	729	99.2	735	99.6	777	93.8	726	98.8	735	100
VACV_TT_002 (ITR)	204	204	100	204	100	204	100	204	100	204	98.5
VACV_TT_003 (ITR)	360	438	82.1	369	96.7	438	81.4	429	83.1	369	96.7	369	97.5	369	95.9	369	97.5
VACV_TT_004 (ITR)	453	360	79.3	360	79.3	363	77.5	597	73.2	453	99.3	438	50.6	597	72.7
VACV_TT_005 (ITR)	549	540	98.2	735	69.1	1134	46.1	1140	45.1	1161	46.9	1275	41.1	1149	47.4
VACV_TT_005.1 (ITR)	492
VACV_TT_006 (ITR)	546	546	99.4	546	98.3	546	100	546	100	546	100	444	97.3	546	99.4
VACV_TT_007 (ITR)	210	210	100	210	100	210	100	210	100	276	65.3	270	68.6	270	68.6
VACV_TT_008 (ITR)	426	423	99.3	426	100	423	98.8	426	99.3	429	97.9	423	97.9	420	96.5	423	97.2
VACV_TT_009 (ITR)	996	996	99.4	996	100	996	100	996	100	996	99.4	996	99.7	996	99.4	996	99.7
VACV_TT_010	198	198	100	198	100	198	100	198	100	198	100
VACV_TT_011	153	252	60.2	252	60.2	363	42.2	252	60.2			543	27.9	252	60.2	546	27.8
VACV_TT_012	189	189	100	189	100	189	100	189	100			201	92.4	189	98.4	189	100
VACV_TT_013	381	381	100	381	95.2	381	97.6	381	100			381	98.4	375	88.9	381	89.7
VACV_TT_014	273	273	100	273	100	273	100	273	100					273	100	714	59.1
VACV_TT_015	429	429	100	429	100	933	42.9	429	99.3			429	99.3	429	99.3
VACV_TT_016	414	192	41.9	426	96.5			399	95.6					408	97.8	414	99.3
VACV_TT_017	234	234	100	234	98.7	234	100	234	100			234	99.6	234	100	234	100
VACV_TT_018	216	216	100	216	100	216	100	216	100					216	100	216	100
VACV_TT_019	180	180	100	180	100	180	100	180	100			180	96.6			183	98.3
VACV_TT_020	1905	1905	100	1905	100	1899	99.7	1905	99.8	1905	99.7	1905	99.6	1905	99.7	1905	99.8
VACV_TT_021	213	213	100	213	100	213	100	213	100	213	98.6
VACV_TT_022	534	534	98.3	534	100	534	98.3	534	99.4	555	92.9	534	99.4	534	97.2	534	98.3
VACV_TT_023	249	249	100	249	100	249	100	249	98.8	249	97.6
VACV_TT_024	225	225	98.6	225	100	225	100	225	98.6	219	91.9
VACV_TT_025	453	453	99.3	453	99.3	453	99.3	453	99.3	453	99.3	453	99.3	453	99.3	453	99.3
VACV_TT_026	456	456	98.7	363	77.5	456	98.7	456	98.7	456	98.7	456	98.7	456	98.7	456	100
VACV_TT_027	615	615	100	615	100	561	91.2	615	100	615	100	615	100	615	100	615	100
VACV_TT_028	594	951	62.5	570	95.9	189	31.7	951	62.5	951	62.3	951	62.3	189	31.7	951	62.3
VACV_TT_028.1						387	63.8							411	68.2
Gene name	TT11	TT8		TT9		TT10		TT12		Cop		ListerV107		Acam 2000		WR
	gene size (bp)	gene size (bp)	similarity vs TT11 (%)	gene size (bp)	similarity vs TT11 (%)	gene size (bp)	similarity vs TT11 (%)	gene size (bp)	similarity vs TT11 (%)	ortholog size (bp)	similarity vs TT11 (%)	ortholog size (bp)	similarity vs TT11 (%)	ortholog size (bp)	similarity vs TT11 (%)	ortholog size (bp)	similarity vs TT11 (%)
VACV_TT_029	792	792	99.2	786	98.1	786	98.5	792	99.6	792	100	792	100	792	100	792	100
VACV_TT_030	210	210	98.6	210	98.6	180	84.8	180	84.8	210	98.6
VACV_TT_031	1539	1539	99.8	1539	100	1539	99.4	1539	100	1539	99.8	1539	99.6	1521	98	1539	99.8
VACV_TT_032	690	675	97.8	675	96.9	675	97.4	675	97.8	675	97.4	675	96.9	675	96.9	690	99.6
VACV_TT_033	354	354	100	354	100	354	100	354	100	354	100	354	100	354	100	354	100
VACV_TT_034	528	528	100	528	99.4	528	100	528	99.4	528	99.4	528	100	528	99.4	528	98.9
VACV_TT_035	1413	1413	100	1413	100	1413	100	1413	100	1419	99.6	1413	100	1410	98.5	1419	99.4
VACV_TT_036	663	663	100	663	100	663	100	663	100	663	100	663	100	663	100	663	99.5
VACV_TT_037	855	855	99.6	855	99.6	855	99.3	855	100	855	98.9	855	99.6	855	100	855	99.6
VACV_TT_038	1110	1110	100	1110	99.5	1110	98.4	1110	99.7	1110	100	1110	98.6	1110	99.5	1110	99.5
VACV_TT_039	246	246	100	246	98.8	246	98.8	246	98.8	246	100
VACV_TT_040	240	240	100	240	100	240	100	240	100	240	100
VACV_TT_041	267	267	100	267	100	267	100	267	98.9	267	100	267	98.9	267	97.7	267	98.9
VACV_TT_042	1275	1275	100	1275	100	1275	100	1275	100	1275	100	1275	100	1275	100	1275	99.8
VACV_TT_043	366	366	100	366	100	366	100	366	100	411	88.2	528	69.1	411	88.2	405	87.3
VACV_TT_044	255	255	100	246	96.4	246	96.4	255	97.6	246	96.4	246	96.4	246	96.4	246	96.4
VACV_TT_045	450	450	99.3	450	99.3	450	99.3	450	99.3	450	99.3	450	99.3	450	99.3	450	99.3
VACV_TT_046	681	723	92.9	681	98.7	681	100	681	98.7	681	100	681	100	681	100	681	99.6
VACV_TT_047	444	444	100	444	100	444	100	444	100	444	100	444	99.3	444	100	444	98.6
VACV_TT_048	1443	1443	100	1443	100	1443	100	1443	100	1443	99.8	1443	100	1443	100	1443	100
VACV_TT_049	960	960	100	960	100	960	99.7	960	100	960	100	960	100	960	99.7	960	100
VACV_TT_050	237	237	100	237	100	237	98.7	237	100	237	98.7
VACV_TT_051	291	291	99	291	99	291	100	291	100	291	99
VACV_TT_052	969	966	99.4	969	100	969	100	969	100	966	99.7	966	99.1	966	98.8	969	99.4
VACV_TT_053	225	225	100	225	100	225	100	225	100	225	100	225	100	225	100	225	100
VACV_TT_054	243	249	97.6	249	97.6	249	97.6	249	97.6	279	85.9	243	100	243	100	243	100
VACV_TT_055	198	198	100	198	98.5	198	100	198	98.5	198	96.9	198	98.5	198	96.9	198	100
VACV_TT_056	639	639	100	639	100	639	100	639	100	639	100	639	100	639	99.5	639	100
VACV_TT_057	1320	1320	100	1320	100	1320	100	1320	100	1320	100	1320	100	1320	100	1320	100
VACV_TT_058	258	258	100	258	100	258	100	258	100	258	100
VACV_TT_059	1065	1065	100	1065	100	1065	100	1065	100	1065	99.7	1065	99.4	1047	98.3	1047	97.7
VACV_TT_060	1908	1908	99.5	1908	99.5	1908	99.8	1908	99.5	1908	99.2	1908	99.4	1908	99.1	1908	99.5
Gene name	TT11	TT8		TT9		TT10		TT12		Cop		ListerV107		Acam 2000		WR
	gene size (bp)	gene size (bp)	similarity vs TT11 (%)	gene size (bp)	similarity vs TT11 (%)	gene size (bp)	similarity vs TT11 (%)	gene size (bp)	similarity vs TT11 (%)	ortholog size (bp)	similarity vs TT11 (%)	ortholog size (bp)	similarity vs TT11 (%)	ortholog size (bp)	similarity vs TT11 (%)	ortholog size (bp)	similarity vs TT11 (%)
VACV_TT_061	216	216	98.6	216	98.6	216	100	216	100	216	98.6
VACV_TT_062	1119	1119	99.7	1119	100	1119	100	1119	100	1119	100	1119	99.7	1119	100	1119	100
VACV_TT_063	222	222	100	222	100	222	100	222	100	222	100	222	100	222	100	222	100
VACV_TT_063.5	150	150	100	150	98	150	98	150	100							150	100
VACV_TT_064	225	225	100	225	98.6			225	98.6	225	98.6
VACV_TT_065	477	477	98.7	477	98.7	477	99.4	477	99.4	477	99.4	477	98.7	477	98.7	444	98.6
VACV_TT_066	696	696	99.6	696	100	696	99.6	696	100	696	99.1	696	99.6	696	99.6	696	99.6
VACV_TT_067	306	306	100	306	100	306	100	306	100	306	100	306	100	306	100	306	100
VACV_TT_068	213	213	100	213	100	213	100	213	100	213	100
VACV_TT_069	1440	1440	100	1440	99.8	1440	99.8	1440	99.6	1440	99.6	1440	99.4	1440	99.4	1440	99.8
VACV_TT_070	2214	2214	100	2214	100	2214	99.9	2214	99.7	2214	100	2214	99.9	2214	100	2214	99.9
VACV_TT_071	573	573	98.4	573	98.9	573	100	573	98.9	573	100	573	97.9	573	98.9	573	98.4
VACV_TT_072	780	780	99.6	780	100	780	99.6	780	100	780	99.6	780	100	780	99.6	780	100
VACV_TT_073	1026	1026	100	1026	99.4	1026	99.7	1026	100	996	98.8	996	99.1	570	55.6	1026	99.1
VACV_TT_074	321	321	100	321	98.1	321	100	321	100	321	98.1
VACV_TT_075	1704	1704	100	1704	99.8	1704	99.8	1704	100	1704	100	1704	100	1704	100	1704	99.8
VACV_TT_076	501	501	99.4	501	100	501	100	501	100	501	100	501	100	501	100	501	100
VACV_TT_077	213	213	98.6	213	100	213	100	213	100	213	100
VACV_TT_078	822	822	99.3	822	100	822	99.6	822	100	822	99.3	822	99.3	822	99.6	822	99.6
VACV_TT_079	201	201	100	201	100	201	100	201	100	201	100
VACV_TT_080	3021	3021	100	3021	100	3021	99.9	3021	100	3021	99.9	3021	100	3018	99.6	3021	99.9
VACV_TT_081	198	198	100	198	100	198	100	198	100	198	100
VACV_TT_082	288	288	100	288	100	288	98.9	288	100	288	98.9	288	100	288	100	288	100
VACV_TT_083	390	390	100	390	100	390	100	390	100	390	100	390	100	390	100	390	100
VACV_TT_084	2001	2001	99.8	2001	99.8	2001	100	2001	99.8	2001	99.8	2001	99.8	2001	99.7	2001	100
VACV_TT_085	348	348	100	348	100	348	100	348	100	348	100
VACV_TT_086	189	189	100	189	100	189	100	189	100
VACV_TT_087	327	327	100	327	100	327	100	327	100	327	100	327	100	327	100	327	100
VACV_TT_088	939	939	100	939	100	939	100	939	100	939	100	939	100	939	99.7	939	100
VACV_TT_089	222	222	100	222	100	222	100	222	100	222	100	222	100	222	98.6	222	100
VACV_TT_090	810	810	100	810	100	810	100	810	100	810	99.6	810	100	810	100	810	100
VACV_TT_091	2316	2316	99.9	2316	100	2316	100	2316	100	2316	99.9	2316	99.9	2316	99.9	2316	100
VACV_TT_092	234	234	100	234	100	234	100	234	100	234	100
VACV_TT_093	240	240	100	240	100	240	100	240	100	240	100	240	100	240	100	240	100
Gene name	TT11	TT8		TT9		TT10		TT12		Cop		ListerV107		Acam 2000		WR
	gene size (bp)	gene size (bp)	similarity vs TT11 (%)	gene size (bp)	similarity vs TT11 (%)	gene size (bp)	similarity vs TT11 (%)	gene size (bp)	similarity vs TT11 (%)	ortholog size (bp)	similarity vs TT11 (%)	ortholog size (bp)	similarity vs TT11 (%)	ortholog size (bp)	similarity vs TT11 (%)	ortholog size (bp)	similarity vs TT11 (%)
VACV_TT_094	1149	1149	99.7	1149	99.7	1149	99.7	1149	99.7	1149	100	1149	100	1149	99.5	1149	100
VACV_TT_095	1272	1272	100	1272	100	1272	100	1272	100	1272	100	1272	100	1272	100	1272	100
VACV_TT_096	2031	2031	100	2031	99.9	2031	99.7	2031	100	2031	99.9	2031	100	2031	99.6	2031	100
VACV_TT_097	1776	1776	99.8	1776	99.8	1776	99.8	1776	99.8	1776	99.7	1776	99.8	1776	99.8	1776	99.7
VACV_TT_098	336	336	100	336	100	336	100	336	100	336	100	336	100	336	100	336	100
VACV_TT_099	663	663	99.1	663	98.6	663	99.5	663	99.5	663	99.5	663	99.5	663	99.5	663	99.5
VACV_TT_100	375	375	100	375	100	375	100	375	99.2	375	99.2	375	100	375	99.2	375	99.2
VACV_TT_101	1305	1305	99.8	1305	99.8	1305	99.8	1305	100	1305	99.8	1305	99.5	1305	99.5	1305	99.3
VACV_TT_101.5	192	192	100	192	100	192	100	192	100	192	100	192	100	192	100	192	100
VACV_TT_102	498	498	100	498	100	498	100	498	100	498	100	498	100	498	100	498	100
VACV_TT_103	1116	1116	100	1116	100	1116	100	1116	100	1116	99.7	1116	100	1116	100	1116	100
VACV_TT_104	399	399	100	399	100	399	100	399	100	399	100
VACV_TT_105	219	219	100	219	100	219	100	219	100	219	100
VACV_TT_106	783	783	100	783	100	783	100	783	100	783	100	783	100	783	100	783	100
VACV_TT_107	1023	1023	100	1023	100	1023	100	1023	100	1023	100	1023	100	1023	100	1023	100
VACV_TT_108	753	753	100	753	100	753	100	753	100	753	100	753	100	753	100	753	100
VACV_TT_109	264	258	96.6	258	96.6	258	96.6	258	96.6	264	100	264	100	258	96.6	264	98.9
VACV_TT_110	1053	1053	100	1053	100	1053	100	1053	100	1053	99.4	1053	100	1053	100	1053	99.7
VACV_TT_111	756	756	100	756	100	756	100	756	100	756	100	756	100	756	100	756	100
VACV_TT_112	387	387	100	387	100	387	100	387	100	387	100	387	100	387	100	387	100
VACV_TT_113	462	462	100	462	100	462	99.3	462	100	462	100	462	99.3	462	100	462	99.3
VACV_TT_114	534	534	99.4	534	100	534	99.4	534	100	534	100	534	99.4	534	100	534	100
VACV_TT_115	1002	1002	100	1002	99.7	1002	100	1002	99.7	1002	99.1	1002	99.1	1002	99.7	1002	99.7
VACV_TT_116	558	558	100	558	100	558	100	558	100	558	100	558	100	558	100	558	100
VACV_TT_117	402	402	100	402	100	402	100	402	100	402	100	402	100	402	100	402	100
VACV_TT_118	3861	3861	99.9	3861	99.9	3861	99.9	3861	99.9	3861	99.9	3861	99.9	3861	99.9	3861	99.9
VACV_TT_119	216	216	98.6	216	100	216	100	216	100	216	98.6
VACV_TT_120	516	516	99.4	516	99.4	516	99.4	516	99.4	516	99.4	516	100	516	100	516	100
VACV_TT_121	570	570	100	570	100	570	100	570	100	570	100	570	99.5	570	100	570	100
VACV_TT_122	975	975	99.7	975	99.7	975	99.7	975	100	975	99.7	975	100	975	100	975	99.1
VACV_TT_123	2388	2388	99.9	2388	99.7	2388	99.9	2388	100	2388	99.7	2388	99.7	2388	99.7	2388	99.9
VACV_TT_124	612	612	100	612	99.5	612	99.5	612	99.5	612	100	612	99.5	612	100	612	99.5
Gene name	TT11	TT8		TT9		TT10		TT12		Cop		ListerV107		Acam 2000		WR
	gene size (bp)	gene size (bp)	similarity vs TT11 (%)	gene size (bp)	similarity vs TT11 (%)	gene size (bp)	similarity vs TT11 (%)	gene size (bp)	similarity vs TT11 (%)	ortholog size (bp)	similarity vs TT11 (%)	ortholog size (bp)	similarity vs TT11 (%)	ortholog size (bp)	similarity vs TT11 (%)	ortholog size (bp)	similarity vs TT11 (%)
VACV_TT_125	945	945	99.7	945	99.7	945	99.7	945	100	945	99.7	945	99.7	945	100	945	99.7
VACV_TT_126	183	183	100	183	100	183	100	183	100
VACV_TT_127	441	441	99.3	441	99.3	441	98.6	441	99.3	441	100	441	100	441	99.3	441	99.3
VACV_TT_128	2535	2535	100	2535	100	2535	100	2535	100	2535	100	2535	99.8	2535	99.9	2535	99.9
VACV_TT_129	255	255	100	255	100	255	100	255	100	255	100
VACV_TT_130	441	441	100	441	100	441	100	441	100	441	100	441	100	441	100	441	99.3
VACV_TT_131	243	243	100	243	100	243	100	243	100	243	100
VACV_TT_132	714	714	100	714	99.2	714	99.6	714	100	714	99.2	714	99.2	714	99.2	714	99.2
VACV_TT_133	657	657	100	657	99.5	657	100	657	100	657	99.1	657	99.5	657	99.5	657	99.5
VACV_TT_134	210	210	98.6	210	98.6	210	98.6	210	98.6	210	95.7
VACV_TT_135	2358	2358	99.9	2358	99.9	2358	99.9	2358	99.9	2358	99.9	2358	99.9	2358	100	2358	99.9
VACV_TT_136	219	219	100	219	100	219	100	219	100	219	100
VACV_TT_137	243	243	100	243	100	243	100	243	100	243	100
VACV_TT_138	1914	1914	99.8	1914	100	1914	100	1914	100	1914	99.7	1914	99.8	1914	99.8	1914	99.8
VACV_TT_139	486	486	100	486	100	486	100	486	100	486	99.4	486	99.4	486	100	486	100
VACV_TT_140	915	915	99.7	915	99.7	915	100	915	99.7	915	99	915	99.3	915	99.3	915	99.3
VACV_TT_141	642	642	100	642	100	642	100	642	100	642	99.5	642	99.5	642	99.5	642	100
VACV_TT_142	747	747	100	747	100	747	100	747	100	747	98.8	747	99.6	747	98.8	747	99.2
VACV_TT_143	1896	1896	100	1896	100	1896	100	1896	100	1896	100	1896	100	1896	100	1896	99.7
VACV_TT_144	210	210	100	210	100	210	100	210	100	210	100
VACV_TT_145	273	273	100	273	100	273	100	273	100	273	100
VACV_TT_146	864	864	100	864	100	864	100	864	100	864	100	864	100	864	100	864	100
VACV_TT_147	219	219	100	219	100	219	100	219	100
VACV_TT_148	1656	1656	99.6	1656	100	1656	99.8	1656	100	1656	99.6	1656	99.6	1656	99.6	1656	99.6
VACV_TT_149	225	225	97.3	225	100	225	97.3	225	97.3	225	100
VACV_TT_150	453	453	100	453	100	453	100	453	100	453	100	453	100	453	100	453	100
VACV_TT_151	675	675	100	675	100	675	100	675	100	675	100	675	100	675	100	675	100
VACV_TT_151.5	231	231	100	231	100	231	100	231	100	231		231	100	231	100	231	98.7
VACV_TT_152	1935	1935	99.7	1935	100	1935	100	1935	99.7	1935	99.7	1935	99.7	1935	99.7	1935	99.7
VACV_TT_153	360	360	99.2	360	100	360	99.2	360	99.2	360	99.2
VACV_TT_154	846	852	98.2	852	97.9	852	97.9	852	97.5	846	98.9	846	98.9	846	97.5	846	98.6
Gene name	TT11	TT8		TT9		TT10		TT12		Cop		ListerV107		Acam 2000		WR
	gene size (bp)	gene size (bp)	similarity vs TT11 (%)	gene size (bp)	similarity vs TT11 (%)	gene size (bp)	similarity vs TT11 (%)	gene size (bp)	similarity vs TT11 (%)	ortholog size (bp)	similarity vs TT11 (%)	ortholog size (bp)	similarity vs TT11 (%)	ortholog size (bp)	similarity vs TT11 (%)	ortholog size (bp)	similarity vs TT11 (%)
VACV_TT_155	261	261	98.8	261	98.8	261	98.8	261	98.8	261	100
VACV_TT_156	495	495	100	495	100	495	100	495	100	495	100	495	100	495	100	495	100
VACV_TT_157	1119	1119	100	1119	100	1119	99.7	1119	100	1119	100	1119	100	1119	100	1119	99.7
VACV_TT_158	2133	2133	99.7	2133	99.9	2133	99.9	2133	100	2133	99.7	2133	99.9	2133	99.9	2133	99.9
VACV_TT_159	387	387	100	387	100	387	100	387	100	387	100
VACV_TT_160	267	267	98.9	267	98.9	267	98.9	267	100	267	97.7
VACV_TT_161	867	867	100	867	100	867	100	867	100	867	100	867	100	867	100	867	100
VACV_TT_162	327	300	90.7	327	100	312	95.4	327	100	300	90.7	300	90.7	327	100	327	100
VACV_TT_163	2679	2679	99.9	2679	99.9	2679	100	2679	99.9	2676	99.3	2676	99.7	2676	99.3	2676	99.7
VACV_TT_164	501	501	99.4	501	100	501	100	501	100	501	98.8
VACV_TT_165	204	231	87.9	231	87.9	231	87.9	231	87.9	228	86.8
VACV_TT_166	228	228	98.7	228	97.3	228	98.7	228	97.3	228	98.7
VACV_TT_167	957	957	100	957	100	957	100	957	100	957	100	957	100	957	100	957	100
VACV_TT_168	576	576	100	576	100	579	99	576	99.5	579	98.4	579	98.4	579	98.4	579	99
VACV_TT_169	213	213	100	213	98.6	213	98.6	213	100	213	97.1	213	100	213	98.6	213	97.1
VACV_TT_170	273	273	100	273	100	273	100	273	100	273	100	273	100	273	100	273	100
VACV_TT_170.5	162	162	100	162	98.1	162	98.1	162	100	162	100	162	100	162	100	162	100
VACV_TT_171	285	285	100	285	100	285	100	285	100	285	100	285	100	285	100	285	100
VACV_TT_172	1137	1137	99.7	1137	99.7	1137	99.7	1137	99.7	1137	99.7	1134	99.5	1137	99.5	1134	99.5
VACV_TT_173	612	612	100	612	100	612	100	612	100	612	100	612	100	612	100	612	100
VACV_TT_174	1482	1482	99.6	1482	99.8	1482	99.6	1482	99.8	1482	99.6	1482	99.6	1482	99.2	1482	99.4
VACV_TT_175	234	234	100	234	100	234	100	234	100	234	100	234	100	234	100	234	100
VACV_TT_176	354	354	99.1	354	99.1	354	100	354	100	354	100	354	100	354	99.1	354	100
VACV_TT_177	1281	1281	99.8	1281	99.8	1281	99.8	1281	99.5	1281	100	1281	99.8	1281	99.8	1281	99.8
VACV_TT_178	387	387	100	387	100	387	100	387	100	387	100
VACV_TT_179	222	222	100	222	100	222	100	222	100	222	100
VACV_TT_180	531	564	93.8	564	93.8	564	93.8	564	93.8	531	100	564	94.1	564	94.1	564	94.1
VACV_TT_181	1149	1149	100	1149	100	1149	100	1149	100	1149	100	1149	99.7	1149	100	1149	99.7
Gene name	TT11	TT8		TT9		TT10		TT12		Cop		ListerV107		Acam 2000		WR
	gene size (bp)	gene size (bp)	similarity vs TT11 (%)	gene size (bp)	similarity vs TT11 (%)	gene size (bp)	similarity vs TT11 (%)	gene size (bp)	similarity vs TT11 (%)	ortholog size (bp)	similarity vs TT11 (%)	ortholog size (bp)	similarity vs TT11 (%)	ortholog size (bp)	similarity vs TT11 (%)	ortholog size (bp)	similarity vs TT11 (%)
VACV_TT_182	3495	3495	99.7	3495	100	3495	99.7	3495	99.8	3495	99.8	3495	99.7	3495	99.8	3495	99.8
VACV_TT_183	222	222	100	222	100	222	100	222	100	222	100
VACV_TT_184	639	639	100			720	49.2	639	100					465	67.6	465	67.6
VACV_TT_185	207	207	100	207	100			207	100
VACV_TT_186	162	162	100	162	100	162	100	162	100
VACV_TT_187	684	693	98.7	693	98.7	684	100	693	98.7					684	99.1	684	99.6
VACV_TT_188	186	186	100	186	100	186	100	186	100
VACV_TT_189	2178	2178	99.6	2178	99.7	2178	99.9	2178	99.6					2166	98.9	2178	99.2
VACV_TT_190	183	183	100	183	100	183	100	183	100
VACV_TT_191	609	1509	40.2	1509	40.2	1509	40.2	1509	40.2	969	60.7	1509	40	1503	40.3	1503	40.6
VACV_TT_192	333	333	100	333	100	333	100	333	100	333	99.1	333	100	333	100	333	100
VACV_TT_193	441	441	100	441	100	441	100	441	100	441	100	441	100	441	100	441	100
VACV_TT_194	918	918	100	918	100	918	100	918	100	918	100	918	100	918	99.7	918	100
VACV_TT_195	213	213	100	213	100	213	100	213	100	213	100
VACV_TT_196	234	234	100	234	100	234	100	234	100	234	100	234	100	234	100	234	100
VACV_TT_197	375	375	100	375	100	375	100	375	100	375	99.2	375	100	375	100	375	100
VACV_TT_198	813	903	89.5	813	98.9	813	100	813	100	903	89	903	89.0	813	99.6	813	98.9
VACV_TT_199	267	267	100	267	100	267	100	267	100	267	100
VACV_TT_200	558	558	99.5	558	100	558	99.5	558	100	558	99.5	558	100	558	99.5	558	99.5
VACV_TT_201	507	507	100	507	100	507	100	507	100	507	99.4	507	100	507	100	507	100
VACV_TT_202	231	231	100	231	100	231	100	231	100	231	100
VACV_TT_203	531	531	100	531	100	531	100	531	100	531	100	531	100	531	100	531	99.4
VACV_TT_204	666	666	100	666	100	666	100	666	100	666	100	666	100	666	100	666	100
VACV_TT_205	792	792	99.2	792	98.9	792	98.9	792	98.9	792	99.2	792	98.9	792	99.2	792	99.2
VACV_TT_206	252	252	100	252	100	252	98.8	252	98.8	252	98.8
VACV_TT_207	195	195	100	195	100	195	100	195	100			189	96.9	189	96.9	189	96.9
VACV_TT_208	183	183	100	183	100	183	100	183	100
VACV_TT_209	834	834	99.3	834	100	834	100	834	100	834	100	834	99.6	834	99.6	834	99.3
VACV_TT_210	774	1212	62.5	687	88.7	687	87.2	774	99.6	1212	62.3	1212	62.5	399	51.2	888	86.1
VACV_TT_210.1	468			429	89.7	468	99.4	468	98.7					429	89	429	89
Gene name	TT11	TT8		TT9		TT10		TT12		Cop		ListerV107		Acam 2000		WR
	gene size (bp)	gene size (bp)	similarity vs TT11 (%)	gene size (bp)	similarity vs TT11 (%)	gene size (bp)	similarity vs TT11 (%)	gene size (bp)	similarity vs TT11 (%)	ortholog size (bp)	similarity vs TT11 (%)	ortholog size (bp)	similarity vs TT11 (%)	ortholog size (bp)	similarity vs TT11 (%)	ortholog size (bp)	similarity vs TT11 (%)
VACV_TT_211	270	270	96.6	270	97.8	270	97.8	270	96.6	270	97.8
VACV_TT_212	480	480	99.4	480	99.4	480	100	480	100	507	88.3	480	100	507	89.6	480	100
VACV_TT_213	660	660	99.1	660	100	660	100	660	100	660	99.5	660	99.5	660	99.1	660	100
VACV_TT_214	402	402	100	402	100	402	100	402	100	402	100	402	100	402	100	402	100
VACV_TT_215	585	585	100	585	99.5	585	99.5	585	99.5	585	99.5	585	99.5	585	99	585	100
VACV_TT_216	237	237	100	237	100	237	100	237	100			237	100			237	100
VACV_TT_217	1041	1041	100	1041	100	1041	100	1041	100	1041	100	1041	99.7	1041	100	1041	99.7
VACV_TT_218	378	378	100	378	100	378	100	378	100	378	99.2	378	99.2	378	100	378	99.2
VACV_TT_219	723	723	100	723	100	723	100	723	100	645	78.2	723	100	723	100	723	100
VACV_TT_220	318	318	100	318	100	318	100	318	100	234	71.4
VACV_TT_221	759	759	100	759	100	759	100	735	96.4	735	96.4	759	99.6	735	96	759	100
VACV_TT_222	684	684	100	684	100	684	100	684	100	615	89.9	684	100	615	89.9	684	100
VACV_TT_223	489	489	100	489	99.4	489	99.4	489	99.4	489	100	489	100	489	99.4	489	100
VACV_TT_224	1659	1659	99.8	1659	100	1659	100	1659	99.5	1659	98.9	1659	98.9	1659	99.6	1659	98.9
VACV_TT_225	201	201	100	201	100	201	100	201	100	201	100
VACV_TT_226	204	204	100	204	100	204	100	204	100	204	100
VACV_TT_227	1005	1005	99.7	1005	99.7	1005	100	1005	100	1005	99.7	801	94.6	801	92.8	1005	100
VACV_TT_228	573	573	100	573	100	573	100	573	100	573	100	573	99.5	573	98.9	573	99.5
VACV_TT_230	561	399	67	399	70.4	561	99.5	399	67	312	49.7	561	100	399	70	312	49.9
VACV_TT_231	180	180	100	180	100	180	100	180	100			180	100
VACV_TT_232	1695	1695	99.6	1695	99.8	1695	100	1695	100	1695	99.6	1695	99.5	1695	99.5	1695	99.5
VACV_TT_233	948	948	99.4	948	99.7	948	99.4	948	99.7	948	99.4	942	98.4	930	96.5	945	97.8
VACV_TT_234	456	456	99.3	456	98.7	456	100	456	99.3	456	99.3	456	99.3	456	98.7	456	98.7
VACV_TT_235	903	903	100	903	100	903	100	903	100	903	99.7	903	99.7	903	100	903	100
VACV_TT_236	219	324	67.3	219	100	219	100	324	67.3	324	67.3
VACV_TT_237	660	660	100	660	98.6	660	99.1	660	100	660	99.1	660	98.2	660	99.5	660	98.6
VACV_TT_238	201	201	100			201	98.5	201	100	201	97
VACV_TT_239	288	288	100	288	100	288	100	288	100	288	100
VACV_TT_240	375	375	100	375	100	375	100	375	100	375	98.4	375	98.4	375	98.4	504	73.1
VACV_TT_241	1677	1677	100	1677	99.6	1677	100	1677	99.8	1677	99.3	1677	99.6	1677	99.5	1677	99.5
Gene name	TT11	TT8		TT9		TT10		TT12		Cop		ListerV107		Acam 2000		WR
	gene size (bp)	gene size (bp)	similarity vs TT11 (%)	gene size (bp)	similarity vs TT11 (%)	gene size (bp)	similarity vs TT11 (%)	gene size (bp)	similarity vs TT11 (%)	ortholog size (bp)	similarity vs TT11 (%)	ortholog size (bp)	similarity vs TT11 (%)	ortholog size (bp)	similarity vs TT11 (%)	ortholog size (bp)	similarity vs TT11 (%)
VACV_TT_242	954	954	99.4	954	99.7	954	99.1	954	100	954	98.7	954	99.7	954	98.7	954	99.4
VACV_TT_243	522	522	100	522	100	522	100	522	100	522	100	522	100	522	99.4	522	100
VACV_TT_244	216	216	100	216	100	216	100	216	100	216	100
VACV_TT_245	549	549	100	549	100	549	100	549	100	549	100	549	100	549	100	549	100
VACV_TT_246	819	819	100	819	100	819	100	816	98.9	819	99.3	819	100	819	100	819	100
VACV_TT_247	174	174	100	174	100	174	100	174	100
VACV_TT_248	234	234	100	234	97.4	234	100	234	100	234	100	234	100	234	100	234	100
VACV_TT_249	501	501	100	501	100	501	100	501	100	501	100	501	98.8	501	100	501	100
VACV_TT_250	219	231	93.1	231	93.1	231	93.5	219	97.7	267	84.1	219	93.3	219	93.3	219	92
VACV_TT_251	852	852	100	852	100	852	100	852	100	852	99.6	852	99.6	852	100	852	99.6
VACV_TT_252	1008	1008	98.5	351	33.6	1008	99.1	1008	98.5	351	32.8	1020	97.2	381	32.5	1038	95.4
VACV_TT_252.1				669	64.2					669	64.5			669	64.5
VACV_TT_253	450	450	99.3	450	99.3	450	100	450	100	450	99.3	450	99.3	450	100	450	100
VACV_TT_254	174	276	63.1	174	100	174	100	174	100	276	62.6
VACV_TT_255	981	981	99.7	981	99.3	981	98.3	981	99.3	873	98.6	981	99.4	981	99.4	981	99.4
VACV_TT_256	228	228	100	228	100	228	100	228	100	228	100
VACV_TT_257	1023	1023	99.1	1023	99.1	1023	99.1	1023	99.1	1023	98.5	1023	99.1	1023	98.8	1023	99.1
VACV_TT_258	1725	1725	99.7	1725	99.7	1725	99.7	1725	99.8	1725	99.5	1242	71.8	1725	99.3	1725	99.8
VACV_TT_259	1056	1056	100	1062	99.4	1056	100	1062	99.4	1062	99.2			798	73.1	1056	100
VACV_TT_260	1794	1809	97.5	1794	99.5	441	24.3	1842	96.4	384	21.1
VACV_TT_261 (ITR)	996	996	99.4	996	100	996	100	996	100					996	99.4	996	99.7
VACV_TT_262 (ITR)	426	423	99.3	426	100	426	100	426	99.3					420	96.5	423	97.2
VACV_TT_263 (ITR)	210	210	100	210	100	210	100	210	100	276	60.2
VACV_TT_264 (ITR)	546	546	99.4	546	98.3	546	100	546	100	546	100	444	97.3	546	99.4
VACV_TT_265 (ITR)	549	540	98.2	735	69.1	1134	46.13	1140	45.1	1161	46.9	1275	41.1	1149	47.4
VACV_TT_265.1 (ITR)	492
VACV_TT_266 (ITR)	453	360	73	360	79.3	363	71.3	597	73.2	453	99.3			597	72.7
VACV_TT_267 (ITR)	360	438	82.1	369	96.7	438	81.4	429	83.1	369	96.7	369	97.5	369	95.9	369	97.5
VACV_TT_268 (ITR)	735	720	98	726	98.8	720	98	729	99.2	735	99.6	777	93.8	726	98.8	735	100
VACV_TT_269 (ITR)	204	204	100	204	100	204	100	204	100	204	98.5

## Sheet: Sheet2

## Sheet: Sheet3
